# A novel treatment strategy for lapatinib resistance in a subset of HER2-amplified gastric cancer

**DOI:** 10.1186/s12885-021-08283-9

**Published:** 2021-08-16

**Authors:** Gang Ning, Qihui Zhu, Wonyoung Kang, Hamin Lee, Leigh Maher, Yun-Suhk Suh, Michael Michaud, Mayerlin Silva, Jee Young Kwon, Chengsheng Zhang, Charles Lee

**Affiliations:** 1 The Jackson Laboratory for Genomic Medicine, 10 Discovery Drive, Farmington, CT 06032 USA; 2 Department of Life Science, Ewha Womans University, Seoul, 03760 Republic of Korea; 3 Department of Molecular and Human Genetics, Baylor College of Medicine, Houston, TX 77030 USA; 4 Department of Surgery, Seoul National University College of Medicine, Seoul, 03080 Republic of Korea; 5 Precision Medicine Center, The First Affiliated Hospital of Xi’an Jiaotong University, 277 West Yanta Rd, Xi’an, 710061 Shaanxi People’s Republic of China

**Keywords:** CRISPR/Cas9 screening, HER2 amplification, Lapatinib resistance, Gastric cancer, PI3K pathway, And MAPK pathway

## Abstract

**Background:**

Gastric cancer (GC) is one of the leading causes of cancer-related deaths worldwide. Human epidermal growth factor receptor 2 (HER2) amplification occurs in approximately 13–23% of all GC cases and patients with HER2 overexpression exhibit a poor prognosis. Lapatinib, a dual EGFR/HER2 tyrosine kinase inhibitor, is an effective agent to treat HER2-amplified breast cancer but it failed in gastric cancer (GC) clinical trials. However, the molecular mechanism of lapatinib resistance in HER2-amplified GC is not well studied.

**Methods:**

We employed an unbiased, genome-scale screening with pooled CRISPR library on HER2-amplified GC cell lines to identify genes that are associated with resistance to lapatinib. To validate the candidate genes, we applied in vitro and in vivo pharmacological tests to confirm the function of the target genes.

**Results:**

We found that loss of function of CSK or PTEN conferred lapatinib resistance in HER2-amplified GC cell lines NCI-N87 and OE19, respectively. Moreover, PI3K and MAPK signaling was significantly increased in CSK or PTEN null cells. Furthermore, in vitro and in vivo pharmacological study has shown that lapatinib resistance by the loss of function of CSK or PTEN, could be overcome by lapatinib combined with the PI3K inhibitor copanlisib and MEK inhibitor trametinib.

**Conclusions:**

Our study suggests that loss-of-function mutations of CSK and PTEN cause lapatinib resistance by re-activating MAPK and PI3K pathways, and further proved these two pathways are druggable targets. Inhibiting the two pathways synergistically are effective to overcome lapatinib resistance in HER2-amplified GC. This study provides insights for understanding the resistant mechanism of HER2 targeted therapy and novel strategies that may ultimately overcome resistance or limited efficacy of lapatinib treatment for subset of HER2 amplified GC.

**Supplementary Information:**

The online version contains supplementary material available at 10.1186/s12885-021-08283-9.

## Background

Gastric cancer (GC) is, worldwide, the fourth most common cancer and the second most common cause of deaths of all malignancies [1, 2]. The American Cancer Society estimated that there would be about 27,510 new GC cases and 11,140 deaths from GC in the US during 2019 [3]. As an oncogenic driver, HER2 gene amplification or HER2 oncoprotein overexpression occurs in approximately 13–23% of GC cases [4]. HER2 amplification and overexpression are associated with poor prognosis in GC patients [5, 6].

HER2 is transactivated through heterodimerization with other HER family members. Notably, HER2 overexpression promotes tumor-cell proliferation, adhesion, migration, and survival via constitutive activation of cascades in the downstream signaling transduction of two pathways: the Ras/Raf/Mitogen-activated protein kinase (MAPK) pathway and the phosphatidylinositol 3 kinase (PI3K)/AKT/mammalian target of rapamycin (mTOR) pathway [7]. HER2-targeted therapies using trastuzumab, a monoclonal antibody, and lapatinib, a small molecule tyrosine kinase inhibitor, have been shown to be effective in treatment of breast cancer patients [8]. However, clinical trial data of HER2-amplified GC have shown that trastuzumab improved overall survival by only 2.7 months, and that lapatinib failed to improve survival in HER2-positive GC patients [9–11]. These unsatisfactory results can be attributed to the intrinsic or acquired resistance to HER2-targeted therapy. To improve the efficacy of HER2-targeted therapy in GC patients, there is an urgent need to elucidate the mechanisms of resistance. Previous studies have suggested that MET and CCNE1 amplifications were involved in lapatinib resistance, by compensating for HER2 via re-stimulation of downstream signaling pathways [12, 13]. Further studies are needed to identify the mechanisms of resistance for patients who do not carry those known genomic alterations.

CRISPR-Cas9 gene editing-based library screening using cell-based assays has been proven to be a very efficient tool for screening for gene mutations that confer drug resistance [14]. This approach is considered to be superior to short hairpin RNA (shRNA) library screening because of its robustness, and its higher specificity and efficiency [15, 16]. To identify genomic alterations potentially associated with lapatinib resistance, we employed a gene-knockout screening approach using a pooled genome-scale CRISPR-Cas9 knockout (GeCKO) V2 library on two HER2-amplified GC cell lines. We identified and validated a set of genes having loss-of-function mutations that contribute to lapatinib resistance. We also demonstrated that the lapatinib resistance involves restoration of the PI3K/AKT and MAPK pathways, and that this restoration is induced by the loss of function of either CSK or PTEN. Moreover, our study shows that the resistance to lapatinib in these HER2-amplified GC cells could potentially be overcome using a combinational treatment with lapatinib; copanlisib, a PI3K inhibitor; and trametinib, a MEK inhibitor. Our findings not only reveal genes and signaling pathways that contribute to lapatinib resistance, but also provides a potential treatment strategy for a subset of HER2-amplified GC.

## Methods

### Cell culture and reagents

Human GC cell lines (N87, OE19) were obtained from the American Type Culture Collection (ATCC). All cell lines were cultured in RPMI1640 medium (Life Technologies) with 10% FBS (Life Technologies), penicillin (100 U/mL; Life Technologies), and streptomycin (100 U/mL; Life Technologies). All cells were maintained in a humidified incubator with 5% CO_2_ at 37 **°**C. Drug treatment reagents lapatinib, trastuzumab, LY294002, saracatinib (AZD0530), rapamycin, trametinib (GSK1120212) and copanlisib (BAY 80–6946), 5- FU were purchased from Selleckchem.

### CRISPR library gene knockout screening

The human GeCKO lentiviral pooled library lentiCRISPR v2 in one plasmid system was purchased from Addgene (Cat # 1000000048) as two half-libraries (library A and library B). Genome-wide loss of function screen using GeCKO library was carried out as described [17]. Briefly, the library plasmid DNA was transformed using electroporation method in Lucigen Endura electrocompetent cells (Lucigen). The grown colonies were recovered from the plates, followed by plasmid DNA extraction using the Endotoxin-Free NucleoBond Xtra Maxi Plus EF kit (Takara). For lentiviral transduction, 293FT cells were co-transfected with lentiCRISPRv2 half-library A or B vector DNA, pCMV-VSVg and psPAX2 (Addgene) using Lipofectamine 2000 and PLUS reagent (ThermoFisher Scientific). After 48 h, supernatants from the transfected 293FT cells were harvested and concentrated using Lenti-X concentrator (Takara) according to the manufacturer’s instructions. Pooled lentiviral libraries are transduced to 1 × 10^8^ GC cells with 3 × 10^6^ cells plated per transduction well. The multiplicity of infection (MOI) is about 0.3 to ensure that most cells receive only one stably integrated RNA guide. Puromycin (1.5 μg/mL for OE19 cells and 0.75μg/ml for N87 cells) was added to the cells at 24 h post transduction and maintained for 7 days. Baseline cells were harvested after puromycin selection. Then transduced GC cells were treated with lapatinib (1 uM for OE19 cells and 0.5 uM for N87 cells) or an equal volume DMSO for 14 days and the survived cells were harvested. For each cell line, two separate infection replicates were performed. The genomic DNA was extracted for PCR amplification and deep sequencing of the genomic regions containing the sgRNAs was conducted. The datasets generated and/or analyzed during the current study are available in the GEO repository (series accession number GSE148668), [https://www.ncbi.nlm.nih.gov/geo/query/acc.cgi?acc=GSE148668].

### Validation of candidate genes

For validation study, selected sgRNAs that target the candidate genes were individually synthesized and cloned into the lentiCRISPR V2 plasmid (Addgene, #52961). Viral particles were generated as described above. Then N87 and OE19 cells were infected with the corresponding viruses and the lapatinib resistance was examined by treating the cells with indicated doses of lapatinib for 6 days. Cell viability assay was performed as described below at the end of treatment.

### Cell viability assay

For the cell viability assays, 4000 cells/each well in a 96-well plate were treated with indicated drugs for 6 days and cell viabilities were measured using the CellTiter-Glo® luminescent cell viability assay kit according to the manufacturer’s instructions (Promega). The luminescence intensity was measured using a multi-label plate reader (SpectraMax M5, Molecular Devices). The cell viabilities were calculated as relative values compared to the untreated controls.

### Western blotting

After indicated treatment, cells were harvested and lysed with RIPA lysis buffer (Thermofisher Scientific) supplemented by protease inhibitor/phosphatase inhibitor cocktails (Cell signaling Technology). Lysates were separated on NuPAGE™ 4–12% Bis-Tris protein gels (Invitrogen) and were transferred to PVDF membranes (Millipore). The membranes were blocked with 5% fat-free milk (Cell signaling Technology) dissolved in TBST buffer (50 mM Tris-HCl, 150 mM NaCl, 0.1% Tween-20). Then, the membranes were incubated with primary antibodies overnight at 4 °C. CSK antibody (#4980), PTEN antibody (#9188), MAPK1/2 antibody (#9102), Phospho-MAPK1/2 (Thr202/Tyr204) antibody (#4370), Phospho-AKT (Ser473) antibody (#9271), AKT antibody (#9272) were purchased from Cell Signaling Technologies. GAPDH antibody (FL-335) was obtained from Santa Cruz biotechnology and horseradish peroxidase-conjugated secondary antibodies (anti-rabbit: NA934V, anti-mouse: NA931V) were purchased from GE healthcare. SuperSignal West Pico Chemiluminescent Substrate (Pierce) was used to detect signals.

### Caspase-Glo 3/7 apoptosis assay

Caspase activity was detected by using Caspase-Glo 3/7 assay kit (Promega). Briefly, The GC cells were seeded in 96-well white luminometer assay plates at a density of 4000 cells per well and incubated at 37 °C. Cells were treated with either lapatinib or vehicle for 48 h. 100ul caspase 3/7 reagents were added to each well and incubated for 1 h on rotary shaker at room temperature. The luminescence intensity was measured using a multi-label plate reader (SpectraMax M5, Molecular Devices).

### In vivo pharmacological assessment with xenograft model

All procedures are performed in the biosafety cabinet in the Biosafety Label 2 (BSL2) procedure room of the animal facility at UConn Health. All work follows the animal protocols approved by both the Institutional Animal Care and Use Committee (IACUC) of UConn Health and The Jackson Laboratory (#101899–0921 and #101660–0920, respectively) and carried out in accordance with the NIH Guide for the Care and Use of Laboratory Animals. Furthermore, this manuscript adheres to the ARRIVE Guidelines for reporting animal research. Six-to-seven-week-old female NOD/SCID/IL-2γ-receptor null (NSG) mice were purchased from the Jackson laboratory (Bar Harbor, ME, stock number 005557). Mice were maintained on a 12-h light/12-h dark cycle at a controlled temperature of 22–23 °C. Water and standard mouse food were freely available. The initial body weight of the animals at the time of arrival was between 19 and 22 g. Mice were allowed to acclimatize to local conditions for 1 week before being injected with tumor cells. Xenografts bearing N87-WT, N87-CSK^−^/^−^ or N87-PTEN^−^/^−^ tumors were induced by injecting wild type N87, CSK or PTEN null N87 cells (5 × 10^6^ per mouse) subcutaneously into the right flank of mice, respectively. The tumors were then measured twice a week using calipers, and the tumor volume in mm^3^ was calculated according to the following formula: (width^2^ × length)/2. Drug treatment was initiated when tumors reached a volume of 150–250 mm^3^. Mice were randomly divided into seven treatment groups consisting of 8–10 mice in each group: 1) vehicle only, 2) lapatinib only, 3) lapatinib plus trametinib, 4) lapatinib plus copanlisib, 5) trametinib plus copanlisib, 6) lapatinib plus trametinib plus copanlisib, 7) 5-FU. Lapatinib were administered via oral gavage at a concentration of 100 mg/kg in 2% DMSO, 30% polyethylene glycol (PEG) 300 (Sigma), 5% Tween 80 (Sigma) in sterile Milli-Q water Monday through Friday. A dose of 50 mg/kg of 5-FU in was given intraperitoneally once weekly. Trametinib were administered by oral gavage at concentration of 0.3 mg/kg in 30% PEG400 and 0.5% DMSO in sterile Milli-Q water Monday through Friday. Copanlisib were administered by intravenous injection at the dose of 1 mg/kg in 20% PEG 400/ acidified water (0.1 N HC, pH 3.5) three times weekly. After 21 days of treatment, the animals were euthanized using a CO_2_ chamber in accordance with the recommendation of the Panel on Euthanasia of the American Veterinary Medical Association, and the tumors were collected from each mouse to measure the weight. Results are presented as mean volumes or weights for each group. Error bars represent the SD of the mean. Statistical calculations were performed using Prism 8 (GraphPad). Statistical analysis to compare tumor volumes in xenograft-bearing mice was performed with 2-way ANOVA. Differences between two groups of tumor mass were assessed by an unpaired Student’s t-test. Differences between groups were considered statistically significant if *p* value < 0.05.

### CRISPR library data processing and initial analysis

Raw FASTQ files were trimmed using customized scripts. To align the processed reads to the library, the designed sgRNA library sequences were assembled into a Burrows-Wheeler index using the Bowtie build-index function [18]. The qualities of fastq files are evaluated using fastqc with options “-Q33 -q 25 -p 50”. Then high quality reads are mapped to the screening library with < 2 bp mismatches using Bowtie, and the raw read counts of sgRNAs from all samples were merged into a count matrix. Next the effects of gene knockout were estimated using three different algorithms: Model-based Analysis of Genome-wide CRISPR-Cas9 Knockout (MAGeCK) Robust Rank Aggregation (RRA) [19], MAGeCK MLE [20] and edgeR algorithms (Fig. S1A) [21]. MAGeCK RRA algorithm builds a mean-variance model to estimate the variance of the read counts, and uses these variance estimations to model the read count changes for each sgRNA in the treatment samples relative to the control samples. The read count changes (sgRNA scores) of all sgRNAs targeting each gene are then ranked and summarized into one score for the gene (gene score), using a modified RRA algorithm. MAeCK MLE initially use the raw table of reads as input, and models the read count of each sgRNA for each sample by a negative binomial random variable and estimates the essentiality of genes in a CRISPR screen via a maximum likelihood approach. The edgeR algorithm uses high-throughtput sequencing counts to detect significantly selected sgRNAs and genes by negative binomial method.

### RNAseq and data analysis

Total RNA was extracted from indicated GC cell lines using RNeasy mini kit (Qiagen). mRNA-Seq libraries for the Illumina Novaseq platform were generated and sequenced at Novogene (California, USA). For the RNAseq raw sequencing reads, we used HISAT2 version 2.2.0 [22] to generate indexes and to map reads to the human genome assembly GRCh37 (hg19). For assembly, we chose SAMtools version 1.7 [23] and the HTSeq version 0.9.1 [24] as the gene-level read counts could provide more flexibility in the differential expression analysis. Both HISAT2 and HTSeq analyses were conducted using the high-performance research computing resources provided by Jackson Laboratory for Genomic Medicine in the Linux operating system. Differential expression and statistical analysis were performed using DESeq2 (release 3.7) in RStudio (version 1.1.447). We used variance stabilizing transformation to account for differences in sequencing depth. *P*-values were adjusted for multiple testing using the Benjamini-Hochberg procedure. A false discovery rate (FDR) adjusted *p*-value < 0.05 was set for the selection of DEGs, with differential expression defined as |log2 (ratio)| ≥ 0.585 (± 1.5-fold) with the FDR set to 5%. Genes were sorted according to their log2-transformed fold-change values after shrinkage in DESeq2 and used for gene set enrichment analysis (GSEA) [25]. Significant gene sets were used to perform the leading-edge analysis. We detected clusters of pathways that shared many leading-edge genes using a community detection algorithm and manually curated these clusters to elucidate the important phenotype-associated pathway groups visualized on the bar plots. Pathway analysis was conducted with the Kyoto Encyclopedia of Genes and Genomes (KEGG) collection of online databases dealing with genomes, enzymatic pathways, and biological chemicals [26].

### Whole exome sequencing (WES) data analysis

For the 103 GC samples in previous publication [27], we downloaded the raw reads from European Nucleotide Archive with accession number PRJEB10531. For the variant analysis using WES data, all sequencing reads were submitted to a quality control check using FASTX-Toolkit (http://hannonlab.cshl.edu/fastx_toolkit/). The phred value 20 was chosen as the minimum threshold for base quality. Following is the alignment of resulting reads to hg19 reference genome with Burrows-Wheeler Aligner (BWA) [28] and Picard (http://broadinstitute.github.io/picard/) was applied for post-alignment procedures as sorting, indexing, and marking duplicates. The alignments were submitted to local realignment around INDELs and base quality score recalibration by using the Genome Analysis Toolkit (GATK) version 3.5. Single nucleotide variants (SNVs) were identified using MuTect2 on the pre-processed sequencing data with default parameters. For Copy Number Variants (CNVs), the XHMM (eXome-Hidden Markov Model) [29] C++ software was run to detection CNVs from exome sequencing data. XHMM includes several key steps in running depth of coverage calculations, data normalization, CNV calling, and statistical genotyping and involves a number of parameters. In our study, we set all parameters to default (minTargetSize: 10; maxTargetSize: 10,000; minMeanTargetRD: 10; maxMeanTargetRD: 500; minMeanSampleRD: 25; maxMeanSampleRD: 200; maxSdSampleRD: 150) for filtering samples and targets, and prepared the data for normalization via XHMM.

### Statistical analysis

Statistical calculations were performed using Prism 8 (GraphPad). Differences between two variables were assessed by an unpaired Student’s *t* test. An observation was considered significant if the *p*-value was less than 0.05.

## Results

### CRISPR library screening identifies candidate genes with loss of function that confers lapatinib resistance in HER2-amplified GC cells

**Fig. 1:**
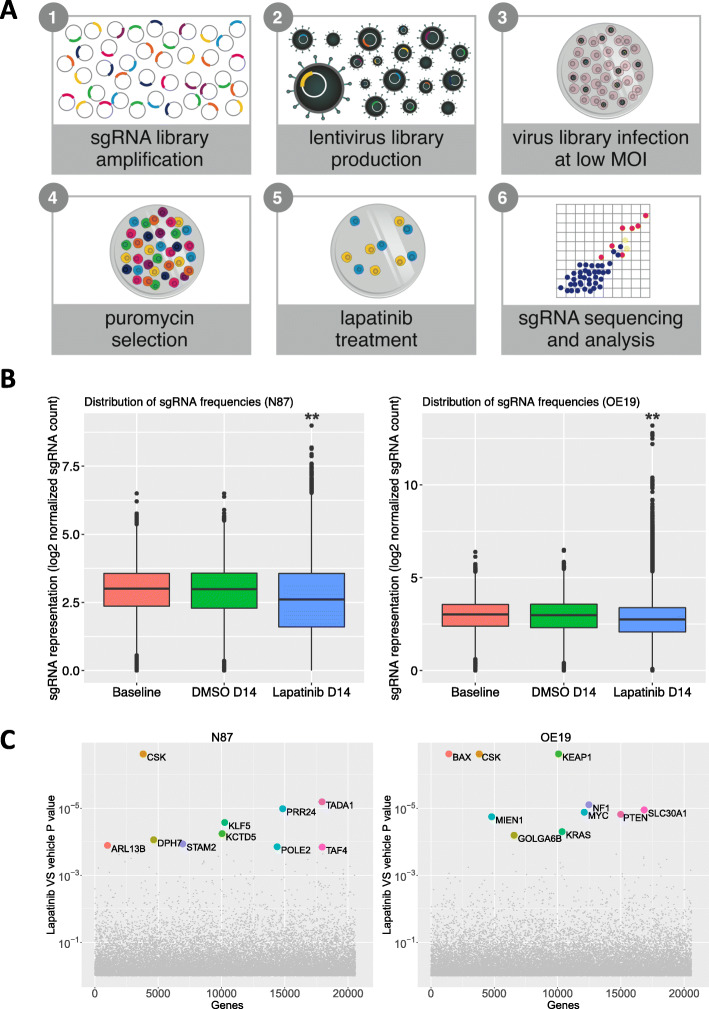
Genome-sale CRISPR library knockout screening for genes associated with lapatinib resistance in GC cell lines. **a** Schematic diagram of the CRISPR library screening strategy. The loss of function screening was performed with the infection of pooled lentivirus containing the GeCKO library V2.0 on N87 and OE19 cells, followed by puromycin selection and lapatinib treatment. The cells were harvested for genomic DNA to PCR the sgRNAs and the subsequent sequencing after 14 days post treatment. **b** The distribution of sgRNA frequencies in the untreated (baseline), the vehicle-treated (DMSO), and lapatinib-treated N87 and OE19 cells, respectively. **c** Scatterplot showing identification of top 10 candidate genes using Model-based Analysis of Genome-wide CRISPR-Cas9 Knockout (MAGeCK). Wilcoxon rank-sum test indicated the following, **significant difference from the control, *p* < 2.2e-16

To identify genes with loss of function that confers drug resistance to lapatinib, we performed a genome-wide CRISPR/Cas9 gene knockout screening in two human GC cell lines harboring HER2 amplification: N87 and OE19. As shown in Fig. 1a, a pooled GeCKO V2 library was amplified for lentivirus production, and then two biological replicates of N87 cells, and two of OE19 cells, were transduced with the lentivirus-containing GeCKO V2 library at a multiplicity of infection (MOI) of 0.3. Puromycin selection was performed before lapatinib treatment on each cell line to enrich successfully transduced cells. After 14 days of lapatinib treatment, the drug-treated and vehicle-treated cells were harvested. Genomic DNA was extracted for PCR amplification and subsequent deep sequencing of the regions containing single-guide RNAs (sgRNAs).

The deep sequencing data showed that the sgRNA distribution in the lapatinib-treated cells was significantly different from that in the vehicle-treated cells, for both the N87 and OE19 cell lines (Wilcoxon rank-sum test, *p*-value < 2.2e-16) (Fig. 1b). The replicates of lapatinib-treated cells are clustered separately from the replicates of cells under other conditions, and all replicates within samples are highly correlated (Pearson correlation coefficient > 0.9) (Fig. S1C), indicating the consistency of our screening system. In addition, we found enrichment of some sgRNAs in the lapatinib-treated cells, by determining, for each sgRNA, the difference in the read count between the lapatinib-treatment samples and the control samples. After 14 days of lapatinib treatment, 694 sgRNAs and three sgRNAs showed greater than 20-fold and 100-fold enrichment, respectively, in the N87 group; and 357 sgRNAs and 34 sgRNAs showed greater than 20-fold and 100-fold enrichment, respectively, in the OE19 group (Table S1 and S2). A panel of candidate genes were detected from this screening using three different algorithms, MAGeCK-RRA, MAGeCK-MLE, and edgeR (Table S3 and S4), and the genes showing the greatest enrichment included CSK, BAX, KEAP1, PRR24, TADA1, KCTD5, PTEN and NF1 which are highlighted shown in Fig. 1c. Our data indicate that loss of these particular genes may contribute to lapatinib resistance.

### In vitro validation study confirms that loss of function of CSK, PTEN*,* and other candidate genes confers resistance to lapatinib

**Fig. 2:**
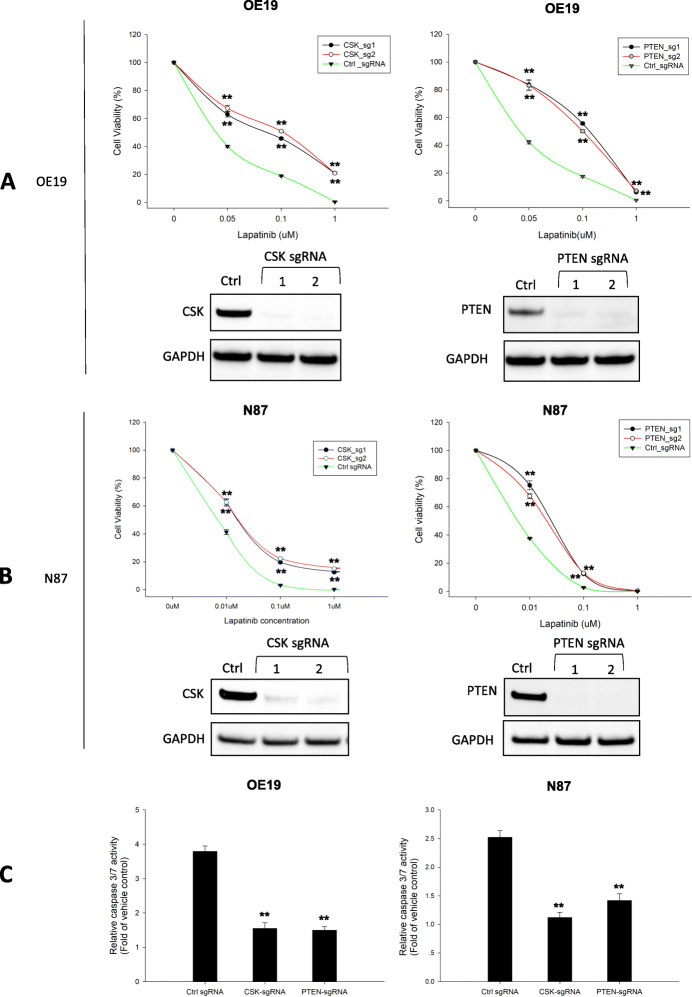
Functional validation of CSK or PTEN null N87 and OE19 cells. **a** Cell viability of CSK or PTEN knockout OE19 cells treated with indicated doses of lapatinib. OE19 cells were transduced with lentiviruses carrying sgRNAs targeting CSK, PTEN, or non-targeting control sgRNA. The drug resistance of the cells from each group was measured by calculating the relative percentage of cell viability. CSK or PTEN protein expressions were evaluated by western blotting. **b** Cell viability of CSK or PTEN knockout N87 cells treated with different doses of lapatinib. N87 cells transduced with non-targeting sgRNA as control. Shift between control cells and gene knockout cells in the dose response curve displays the reduced sensitivity to lapatinib in the GC cell lines. CSK or PTEN protein expressions were evaluated by western blotting. **c** Caspase-Glo 3/7 assay analysis to examine lapatinib induced caspase-3/7 activity after 48 h treatment in CSK or PTEN null cells OE19 and N87 cells. OE19 or N87 cells transduced with virus carrying non-targeting sgRNA as control. Unpaired Student’s *t* test indicated the following: *significant difference from the control, *p* < 0.05; **significant difference from the control, *p* < 0.01

After identifying candidate genes from the screening described above, we performed validation experiments with selected genes to confirm whether or not their loss of function confers lapatinib resistance in GC cells. The genes selected for validation include: 1) genes that were identified as among the top 20 candidates by at least two of the three algorithms (MAGeCK-RRA, MAGeCK-MLE, and edgeR); and 2) genes that were identified as among the top 20 candidates in both N87 and OE19 cells. The genes selected using these criteria are highlighted in gray in Tables S3 and S4. For each gene selected for validation, we selected two sgRNAs targeting that gene. Then, for each gene-targeting sgRNA, N87 and OE19 cells were infected with lentivirus carrying that sgRNA and were then treated with different doses of lapatinib for six days. After treatment, drug resistance was assessed by determining cell viability. CSK, PTEN, KCTD5, MED24, KEAP1, NF1, BAX, TADA1 and KRAS were validated to confer resistance (*p* < 0.05, Tables S3 and S4; validated genes are shown in red). While significant resistance to lapatinib was validated in OE19 cells with loss of function of BAX, KEAP1, NF1, and TADA1, and in N87 cells with loss of function of NF1 and KCTD5, (Fig. S2A and Fig. S2B), our data show that loss of CSK or PTEN in N87 and OE19 cells (confirmed by western blotting) conferred the most significant resistance to lapatinib treatment (OE19 validation groups, two-way ANOVA, ****, *P* < 0.0001, *n* = 3 replicates; N87 validation groups, two-way ANOVA, ***, *P* < 0.001, n = 3 replicates) (Fig. 2a and b). Both CSK and PTEN are tumor-suppressor genes. *CSK* is a non-receptor protein tyrosine kinase that serves as an indispensable negative regulator of the Src family tyrosine kinases (SFKs). Induction of signaling pathways such as STAT3 or PI3K pathway via up-regulation of SRC signaling has been linked to cancer progression [30]. PTEN is a protein tyrosine phosphatase that negatively regulates the PI3K/AKT pathway to repress tumor-cell growth and survival. Because loss of CSK or PTEN exhibited the most significant resistance to lapatinib in both N87 and OE19 cells, we subsequently focused on characterization of CSK-null and PTEN-null cells in this study.

We examined apoptosis induced by lapatinib treatment in CSK- and PTEN-null cells as well as in control cells (cells transduced with non-targeting sgRNA, and cells treated with vehicle) by measuring caspase-3/7 activation (Caspase-Glo 3/7 assay). Consistent with our cell viability results, OE19 cells and N87 cells deficient for CSK or PTEN showed significantly lower levels of apoptosis compared to control cells. OE19 cells transduced with non-targeting sgRNA showed 3.79 ± 0.16-fold greater levels of caspase-3/7 activity compared with vehicle-treated OE19 cells, while CSK- and PTEN-null OE19 cells showed 1.55 ± 0.16-fold and 1.50 ± 0.10-fold greater levels of caspase-3/7 activity, respectively, compared to control cells (control vs CSK-sgRNA, control vs PTEN-sgRNA, unpaired t test: *p* < 0.001) (Fig. 2c). Experiments using CSK- or PTEN-null N87 cells showed similar results (control vs CSK-sgRNA, unpaired t test: *P* < 0.001; control vs PTEN-sgRNA, unpaired t test: *P* < 0.01) (Fig. 2c). Together, these results indicate that loss of function of CSK or PTEN significantly inhibits lapatinib-induced apoptosis.

### Up-regulation of PI3K and MAPK signaling was observed in CSK-null and PTEN-null GC cells

**Fig. 3:**
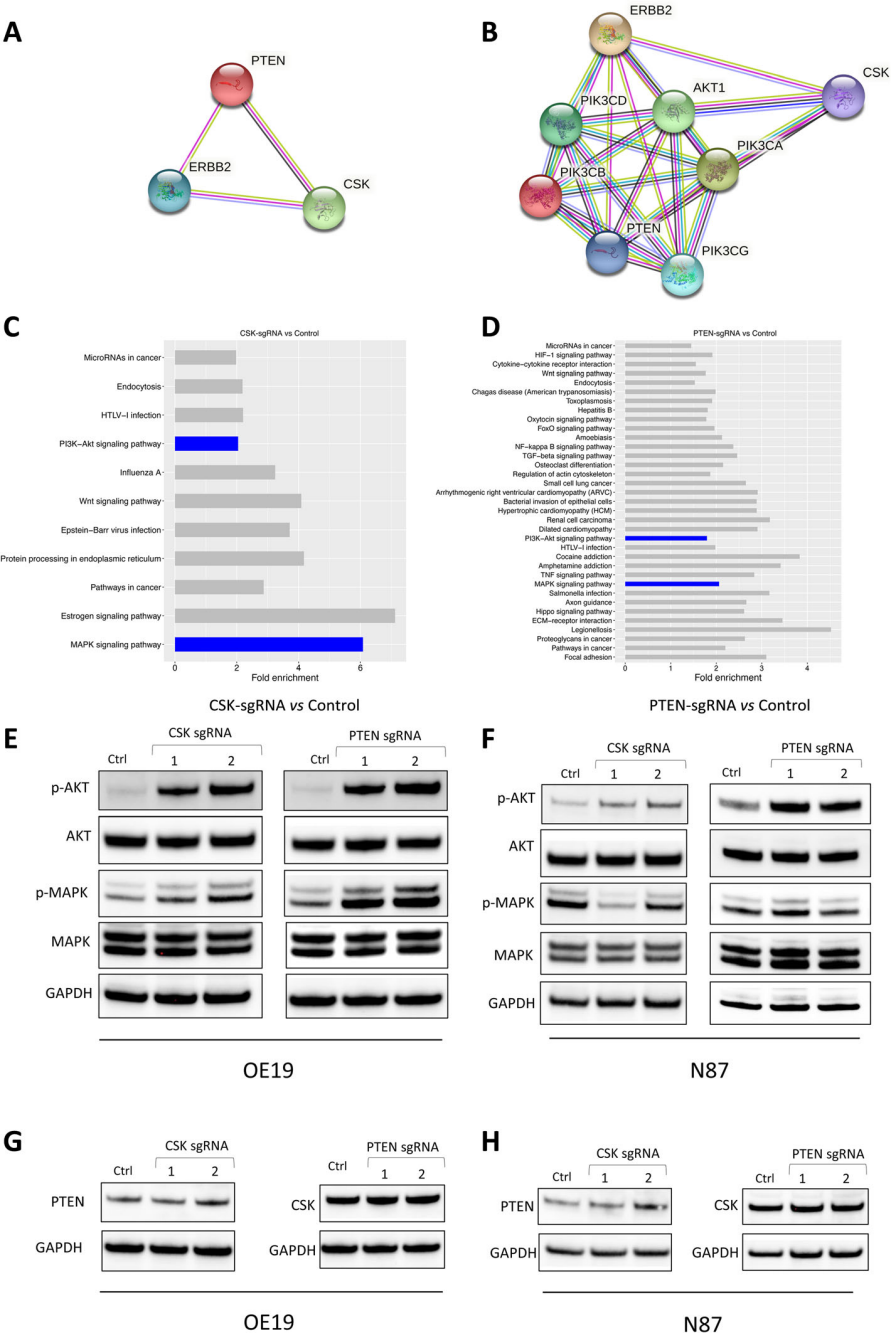
Protein interaction network prediction, gene expression profile and pathway analysis of CSK or PTEN knockout cell lines and Up-regulation of PI3K/AKT and MAPK pathways in the CSK or PTEN knockout GC cells. Protein interaction network (**a**) and the predicted partners (**b**) were analyzed by STRING. CSK, PTEN and HER2 (ERBB2) were mapped by searching the STRING database version 10.5. In the resulting protein association network, proteins are presented as nodes which are connected by lines whose thickness represents the confidence level. (**c**) The bar plot depicts the enriched pathway among the DEGs between CSK null cells (N87-CSK-sgRNA) and parental N87 cells (N87) by KEGG pathway analysis. **d** The bar plot depicts the enriched pathway among the DEGs between PTEN null cells (OE19-PTEN-sgRNA) and parental OE19 cells (OE19) by KEGG pathway analysis. **e** The levels of phosphorylated and total proteins of AKT and MAPK were assessed by western blotting in CSK or PTEN knockout OE19 cells after treating with 0.05 uM lapatinib, respectively. OE19 cells transduced with non-targeting sgRNA as control. **f** The levels of phosphorylated and total proteins of AKT and MAPK were assessed by western blots in CSK or PTEN knockout N87 cells after treating with 0.01 um lapatinib, respectively. N87 cells transduced with non-targeting sgRNA as control. **g** PTEN and CSK protein expression was examined by western blotting in CSK knockout OE19 cell lines and PTEN knockout OE19 cell lines, respectively. OE19 cells transduced with non-targeting sgRNA as control. **h** PTEN protein and CSK protein expression was examined by western blotting in CSK knockout N87 cell lines and PTEN knockout N87 cell lines, respectively. N87 cells transduced with non-targeting sgRNA as control

The similar resistance phenotypes of *CSK* and *PTEN* knockout cells suggest that these two genes might be functionally linked. To further understand whether there is a functional interaction between *CSK* and *PTEN*, we analyzed the interaction networks between the two proteins using Search Tool for the Retrieval of Interacting Genes (STRING) [31]. The networks established by interacting proteins are helpful in elucidating potential molecular mechanisms, including functionally related proteins within the networks, underlying lapatinib resistance in HER2-amplified GC cells. Here, our STRING analysis suggests that *CSK*, *PTEN*, and *ERBB2* (*HER2*) are functionally linked (Fig. 3a), and PI3K/AKT pathway components *PIK3CA*, *AKT1*, *PIK3CG*, *PIK3CB*, and *PIK3CD* are predicted as directly linked functional partners of the three functionally linked proteins (Fig. 3b), indicating that *CSK* and *PTEN* are involved in HER2 signaling and in the PI3K/AKT pathway in GC cells. To better understand the molecular mechanisms of lapatinib resistance in CSK-null and PTEN-null GC cells, we performed RNA-Seq on CSK-null and PTEN-null cells and the corresponding parental cells and compared their transcriptome profiles. The differentially expressed genes (DEGs) are shown in heat maps (Fig. S3C and S3D). A total of 139 genes were identified as significant DEGs between CSK-null cells and parental (N87) cells, and 997 genes were identified as significant DEGs between PTEN-null cells and parental (OE19) cells (for all significant DEGs, fold change > 1.5, FDR < 0.1). The data also suggests that, in GC cells, a mutation resulting in loss of PTEN function has a much greater impact on the gene expression profile compared to a mutation resulting in loss of CSK function. To provide insight into the cellular pathways associated with *CSK* and *PTEN*, we performed Kyoto Encyclopedia of Genes and Genomes (KEGG) pathway analysis of the DEGs. Among the pathways identified as enriched (Fig. 3c and d), the MAPK, PI3K, and Wnt pathways were enriched in both CSK- and PTEN-null GC cells (> 1.5-fold enrichment), suggesting that these pathways play important roles in lapatinib resistance.

Considering that PI3K and MAPK are the major downstream pathways of the HER2 receptor, we hypothesized that loss of CSK or PTEN function would restore the PI3K/AKT and/or MAPK signaling pathways, resulting in resistance to lapatinib. To test this, we examined the phosphorylation levels of AKT and MAPK in CSK-null and PTEN-null GC cells, using western blotting, following treatment of the cells with lapatinib. Consistent with the results of our protein interaction and pathway analyses, we found that, following treatment of OE19 cells with 0.05 μM lapatinib, the phosphorylation levels of AKT and MAPK were substantially higher in CSK- and PTEN-null cells than in the control cells (Fig. 3e). Treatment of N87 cells with 0.01 μM lapatinib yielded a similar pattern of AKT phosphorylation in CSK- and PTEN-null cells compared to the control cells, but the phosphorylation levels of MAPK appear to show little or no difference between, on the one hand, the CSK- or PTEN-null cells, and, on the other, the control cells (Fig. 3f). To determine whether expression of PTEN is dependent on CSK expression, and vice versa, we assessed PTEN expression in CSK knockout OE19 and N87 cells, and CSK expression in PTEN knockout OE19 and N87 cells, by western blotting. Results showed no significant change in expression of either gene as a result of knockout of the other, in either cell line (Fig. 3g and h), suggesting that neither CSK nor PTEN regulates the protein expression of the other.

### Pharmacological inhibition of the PI3K and MAPK pathways synergistically overcomes the resistance to lapatinib in CSK-null and PTEN-null GC cells

**Fig. 4:**
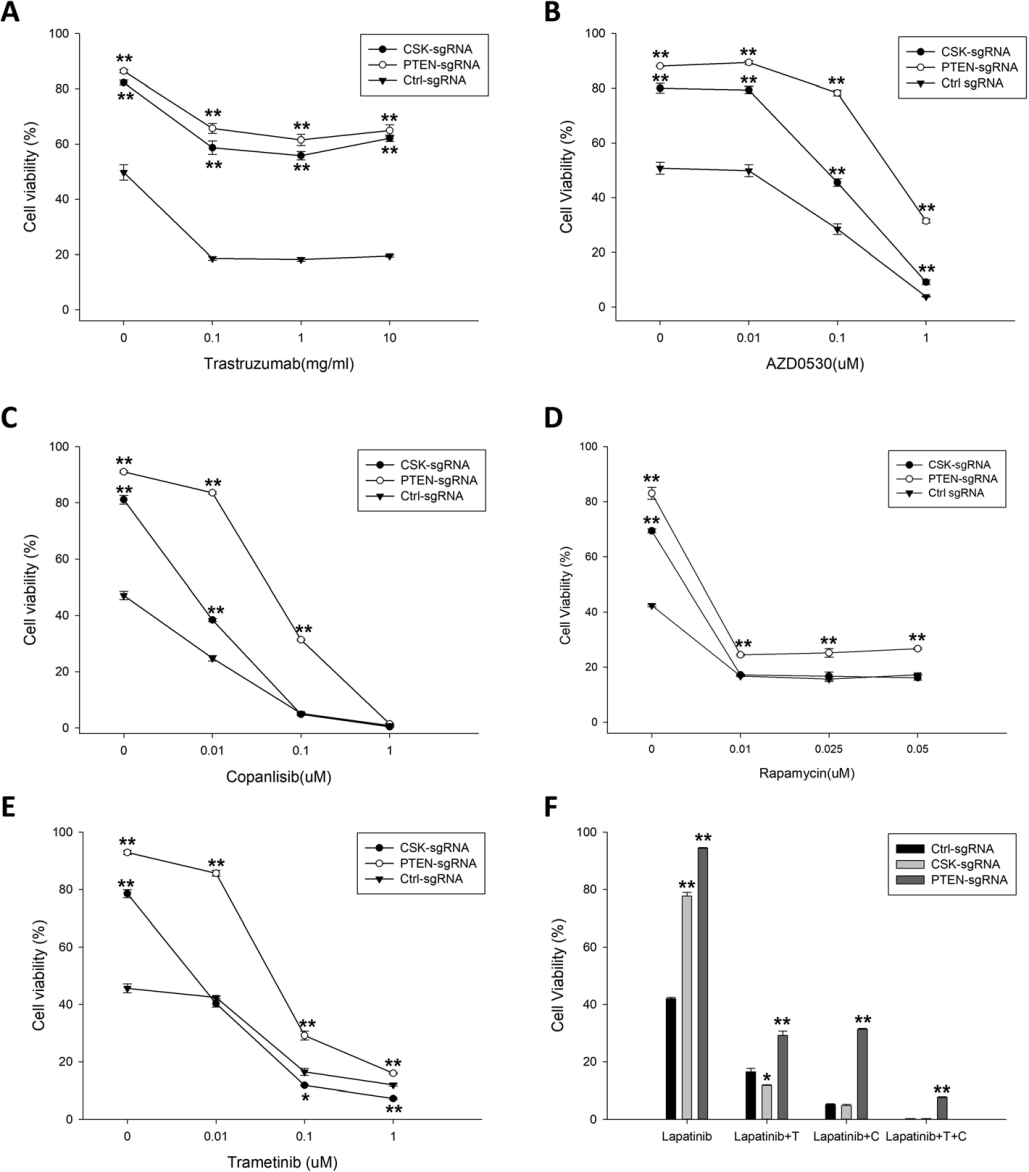
Pharmacological inhibition of PI3K, MAPK and SRC signaling pathways re-sensitizes resistant GC cells to lapatinib. OE19 cells transduced with CSK targeting sgRNAs or PTEN targeting sgRNAs were used for following test. OE19 cells transduced with non-targeting sgRNA as control. **a** Growth curve of test groups with 0.05 μM lapatinib in combination with indicated dose of trastuzumab for 6 days. **b** Growth curve of test groups treated with indicated dose of SRC inhibitor AZD0530 for 6 days. **c** Growth curve of test group treated with 0.05 μM lapatinib in combination with indicated dose of PI3K inhibitor copanlisib for 6 days. **d** Growth curve of test groups treated with 0.05 μM lapatinib in combination with different doses of mTOR inhibitor rapamycin for 6 days. **e** Growth curve of test groups treated with 0.05 μM lapatinib in combination with different doses of MEK inhibitor trametinib for 6 days. **f** Inhibition effect of 0.05 μM lapatinib alone or in combination with 0.1 μM trametinib or /and 0.1 μM copanlisib for 6 days. Unpaired Student’s *t* test indicated the following: *significant difference from the control, *p* < 0.05; **significant difference from the control, *p* < 0.01

Trastuzumab is a potent anti-HER2 agent and is usually applied with or without lapatinib in HER2-amplified breast cancer patients. In addition, a trastuzumab-based treatment has been approved by the FDA as a target treatment for HER2-positive advanced GC [32]. Here, we tested whether loss of CSK or PTEN in GC cells confers resistance to combined trastuzumab and lapatinib treatment. We observed that CSK-null and PTEN-null OE19 cells were significantly more resistant than control cells to combined lapatinib (0.05 μM) and trastuzumab (0.1–10 mg/ml) treatment (Fig. 4a). Similar results were obtained in CSK-null and PTEN-null N87 cells (Fig. S4A), indicating that loss of function of CSK or PTEN confers resistance to both lapatinib and trastuzumab in HER2-amplified GC cells.

To further test our hypothesis that re-activation of the signaling downstream of HER2 is the mechanism underlying the lapatinib resistance, we employed a pharmacological approach to modulate the PI3K/AKT/mTOR, MAPK, and SRC signaling pathways. Because CSK negatively regulates SRC signaling, we first treated CSK-null and PTEN-null OE19 cells with the SRC family kinase inhibitor AZD0530 (saracatinib). As shown in Fig. 4b, AZD0530 (0.01–1 μM) in combination with 0.05 uM lapatinib, decreased the cell viability of CSK- and PTEN-null OE19 cells in a dose-dependent manner, indicating that re-activation of the SRC signaling pathway in both CSK- and PTEN-null OE19 cells may confer the resistance to lapatinib. However, at the highest dose of AZD0530 (1 μM), the percentage of PTEN-null cells that survived the treatment was significantly larger than the percentage of CSK-null cells that survived (42.49 ± 2.54% of PTEN-null cells versus 9.15 ± 0.88% of CSK-null cells, Fig. 4b), suggesting that SRC signaling is the major re-activated pathway in the CSK-null OE19 cells but not in the PTEN-null OE19 cells. We also treated the CSK- and PTEN-null OE19 cells with the PI3K inhibitor copanlisib (BAY 80–6946), a drug approved by the FDA for patients with relapsed follicular lymphoma [33]. Remarkably, copanlisib (0.01–1 μM) in combination with 0.05 μM lapatinib inhibited the growth of CSK- and PTEN-null OE19 cells as well as control cells in a dose-dependent manner (Fig. 4c), and similar results were obtained with another PI3K inhibitor, LY294002 (Fig. S4B), indicating that the PI3K pathway plays an important role in lapatinib resistance of CSK- and PTEN-null GC cells. Because mTOR is a key molecule downstream of the PI3K pathway that regulates cell growth, proliferation, and survival, we tested whether an mTOR inhibitor, rapamycin, could overcome the lapatinib resistance in CSK-null and PTEN-null GC cells. We found that rapamycin (0.01 μM) in combination with lapatinib (0.05 uM) significantly inhibited growth of CSK- and PTEN-null OE19 cells as well as of control cells (Fig. 4d). However, increased doses of rapamycin (0.025–0.05 μM) with 0.05 uM lapatinib did not further decrease cell growth (Fig. 4d), suggesting that mTOR may contribute only partially to lapatinib resistance in the GC cells. In addition, we treated CSK- and PTEN-null OE19 cells with lapatinib and the MAPK inhibitor trametinib (GSK1120212), a drug approved by the FDA in combination with dabrafenib for treatment of patients with BRAF V600E/K-mutant metastatic melanoma [34]. In our pharmacological test, we observed that trametinib (0.01–1 μM) in combination with 0.05 μM lapatinib dramatically overcame the resistance to lapatinib in the CSK- and PTEN-null OE19 cells in a dose-dependent manner (Fig. 4e), indicating that re-activation of the MAPK pathway in CSK- and PTEN-null OE19 cells may also play an important role in lapatinib resistance.

While the combination of lapatinib plus copanlisib or lapatinib plus trametinib could significantly inhibit the lapatinib resistance in the CSK or PTEN null OE19 cells, it requests high dose of drugs (1 μM copanlisib or 1 uM trametinib) to overcome the resistance (Fig. 4c and e). Therefore, we tested another treatment strategy using three drugs combination of lapatinib, copanlisib and trametinib with relative low doses. As shown in Fig. 4f, lapatinib alone didn’t inhibit the growth of CSK or PTEN null OE19 cells, whereas lapatinib plus 0.1 μM copanlisib or lapatinib plus 0.1 μM trametinib significantly inhibited the cell growth, but not completely. When the cells were treated with a combination of these three agents (i.e. 0.05 μM lapatinib, 0.1 μM copanlisib, and 0.1 μM trametinib), the cell viability dramatically decreased to 0.22 ± 0.08% in CSK null OE19 cells and 7.65 ± 0.31% in the PTEN null OE19 cells, respectively. It almost completely overcame the lapatinib resistance. Similar result was presented in CSK or PTEN null N87 cells (Fig. S4C). This finding further supports that re-activation of PI3K and MAPK are both involved in lapatinib resistance in HER2-amplified GC with loss of function of CSK or PTEN. Thus, our study provides a potential therapeutic strategy for the GC patients of HER2 amplification with CSK or/and PTEN loss of function mutation.

### In vivo pharmacological assessment confirms the efficacy of the new therapeutic strategy

**Fig. 5:**
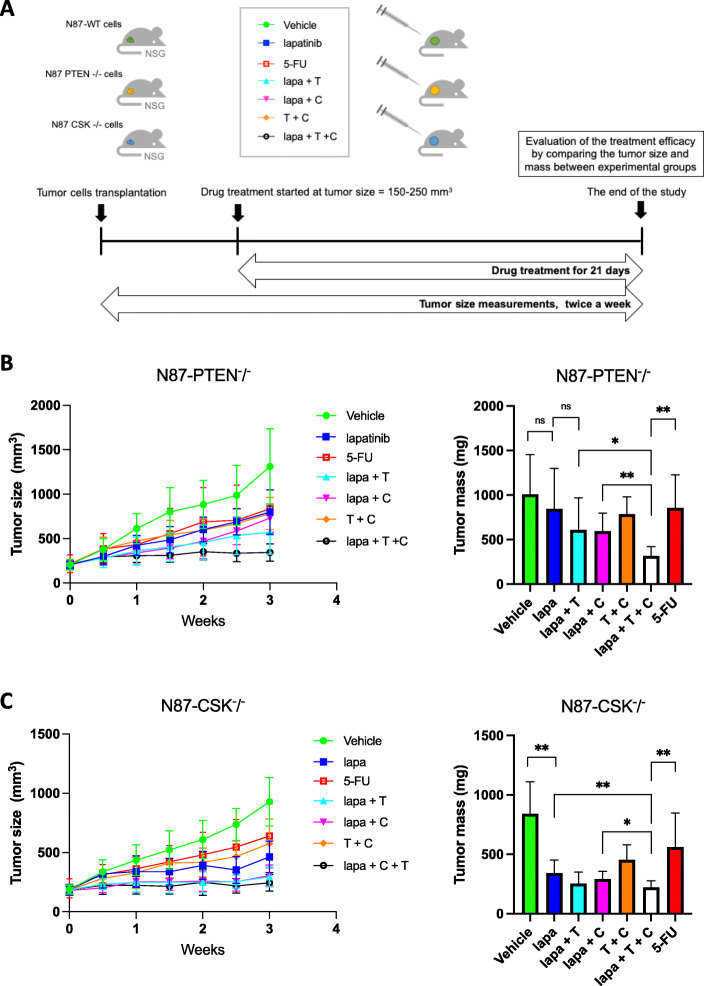
In vivo antitumor efficacy of combination of lapatinib, trametinib and copanlisib comparing with other treatment conditions including vehicle, lapatinib (100 mg/kg), lapatinib plus trametinib (0.3 mg/kg), lapatinib plus copanlisib (1 mg/kg), trametinib plus copanlisib, and 5-FU (50 mg/kg). Tumor volumes of these xenografts are shown on the left and their tumor mass after 3 weeks of treatment are shown on the right. **a** A schematic diagram for in vivo drug treatment experiment with mouse xenograft model. **b** The synergistic effect of trametinib, copanlisib and lapatinib in N87-PTEN^−^/^−^ gastric cancer xenograft model comparing with other treatment conditions. Tumor sizes were measured twice a week and tumor masses were measured at the end of experiment. Tumor volume and mass were presented as mean. Error bars, SD. **c** The antitumor effect of trametinib, copanlisib and lapatinib in N87-CSK^−^/^−^ gastric cancer xenograft model comparing with other treatment conditions. Tumor sizes were measured twice a week and tumor masses were measured at the end of experiment. Tumor volume and mass were presented as mean. Error bars, SD. Unpaired Student’s *t* test indicated the following: *significant difference between the indicated two groups, *p* < 0.05; **significant difference between the indicated two groups, *p* < 0.01

To further validate the efficacy of the drug combination of lapatinib, copanlisib and trametinib, we applied it to in vivo xenograft model for pharmacological assessment (Fig. 5a). Firstly, we did lapatinib dosing test with xenograft models bearing N87-WT, N87-CSK^−^/^−^ and N87-PTEN^−^/^−^ tumors, and found that N87-WT tumors grow relatively slow and it is sensitive to lapatinib treatment (vehicle vs lapatinib 50 mg/kg, 2-way ANOVA: ***, *P* = 0.0009). N87-CSK^−^/^−^ and N87-PTEN^−^/^−^ tumors grow much faster and form big tumor masses after 3 weeks. Comparing with N87-WT tumors, N87-CSK^−^/^−^ tumors are relatively insensitive to lapatinib (vehicle vs lapatinib 100 mg/kg, 2-way ANOVA: ns, *P* = 0.0924) and N87-PTEN^−^/^−^ tumors are resistant to lapatinib treatment (vehicle vs lapatinib 200 mg/kg, 2-way ANOVA: ns, *P* = 0.1607) (Fig. S5). In the second experiment, we compared the efficacy of the drug combinations: lapatinib plus trametinib plus copanlisib and other treatment conditions including gastric cancer standard chemotherapy agent fluorouracil (5-FU). Since N87-WT is sensitive to lapatinib treatment, we exclude it in the drug combination treatment. From the in vivo test with N87-PTEN^−^/^−^ xenograft tumor, a significant effect upon tumor growth was observed with combination of lapatinib, trametinib and copanlisib when compared with vehicle (2-way ANOVA: ****, *P* < 0.0001), lapatinib alone (2-way ANOVA: ***, *P* = 0.0002), 5-FU treatment group (2-way ANOVA: **, *P* = 0.0097), lapatinib plus copanlisib (2-way ANOVA: **, *P* = 0.0067), trametinib plus copanlisib (2-way ANOVA: ****, *P* < 0.0001), respectively (Fig. 5b). Similarly, when the mass of the tumors at endpoint were compared, the three drugs combination showed significant improvement over all other groups (Unpaired t-test: *P* < 0.05) (Fig. 5b). Similar result was obtained from the experiment with N87-CSK^−^/^−^ xenograft (Fig. 5c), except trametinib and copanlisib in the three drug combinations showed synergistic effects in xenografts bearing N87-PTEN^−^/^−^ but not N87-CSK^−^/^−^, indicating the three drugs combination tends to be more effective on more resistant tumors. Overall, the in vivo drug treatment study further suggests that synergistically inhibiting PI3K and MAPK pathways can significantly inhibit tumor growth in those lapatinib resistant tumors with loss of function mutations of CSK or PTEN.

## Discussion

Molecular targeted therapy has shown great specificity in eliminating malignant cells with minimal side effects in cancer therapy compared with conventional chemotherapy [35]. Lapatinib, a dual EGFR and HER2 inhibitor, are clinically effective against HER2-amplified breast cancer by blocking HER2 phosphorylation, resulting in inhibition of downstream PI3K/AKT and MAPK pathways [36]. However, it didn’t improve survival significantly in clinical trials of HER2-amplified gastric cancer (GC), indicating additional oncogenic alterations or the specific tumor microenvironment could contribute to the drug resistance in GC. The previous studies in GC suggest that signaling through other receptor tyrosine kinases (RTKs), such as amplification of MET, IGFR, and HER3 confer anti-HER2 treatment resistance by re-stimulating downstream of PI3K and MAPK signal transduction, thus bypassing the inhibitory effect of lapatinib or trastuzumab [37, 38]. In our CRISPR/Cas9 based genome-wide knockout screening study, we identified and demonstrated that loss of function mutations of CSK or PTEN conferred resistance to lapatinib in HER2-amplified GC cell lines by restoring downstream PI3K and MAPK pathways of HER2 receptor. Interestingly, a previous study in breast cancer suggests that increased SRC kinases activates the PI3K signaling cascade via altering the capacity of the PTEN C2 domain binding to the cellular membranes rather than directly interfering with PTEN enzymatic activity [39]. Combining with our observation, it indicates CSK, the SRC family kinases negative regulator, functionally linked with PTEN in regulating PI3K signaling in lapatinib resistance, which explained the similar resistance phenotype of CSK and PTEN null GC cells and stable PTEN protein level in CSK null cells in our study (Fig. 2 and Fig. 3e-h). In addition, our result is consistent with the previous study in breast cancer that loss of function of PTEN triggered hyperactivation of the MAPK pathway [40]. Taken together, our data suggest that loss of function mutation of CSK or PTEN leads to the up-regulation and hyperactivation of PI3K and MAPK pathways, which could be the central mechanism for lapatinib resistance in these GC cells.

Furthermore, our in vitro and in vivo pharmacological study showed significant synergistic effect of PI3K inhibitor copanlisib and MEK inhibitor trametinib to inhibit resistant GC tumor growth when they are combined with lapatinib (Figs. 4 and 5). This finding could be potentially important for developing novel anti-HER2 therapy. In particular, HER2-amplified GC patients with CSK or PTEN mutation might therefore be good candidates for combinational therapy with lapatinib, PI3K inhibitor and MEK inhibitor. To explore the potential clinical application, we checked the status of PTEN or CSK mutations in the HER2-amplified GC cases. For this purpose, we collected the variants data from over 630 GC patient samples from the public resource (cBioPotal) [41], and 103 GC patient samples from our previous study [27]. Over 23% of GC patients showed HER2 amplification in the public samples. Approximately 14% of GC patients harbored HER2 amplification in our dataset. 3–14% of the GC patients with HER2 amplification have either PTEN or CSK mutations in the genome (Table S5). Of note, the public cohort data is only based on somatic mutations and this percentage could be higher if we include germline mutations. In addition, GC patients with HER2 amplification and gene alterations associated with PI3K or MAPK pathways could also benefit from this treatment strategy. These gene alterations include but not limited to PIK3CA, PIK3CB, EGFR, KRAS, BRAF and MLK3. For example, gain of function mutations in PIK3CA have been suggested to be associated with trastuzumab/lapatinib resistance by up-regulating PI3K pathway in breast cancer [42, 43].

**Fig. 6:**
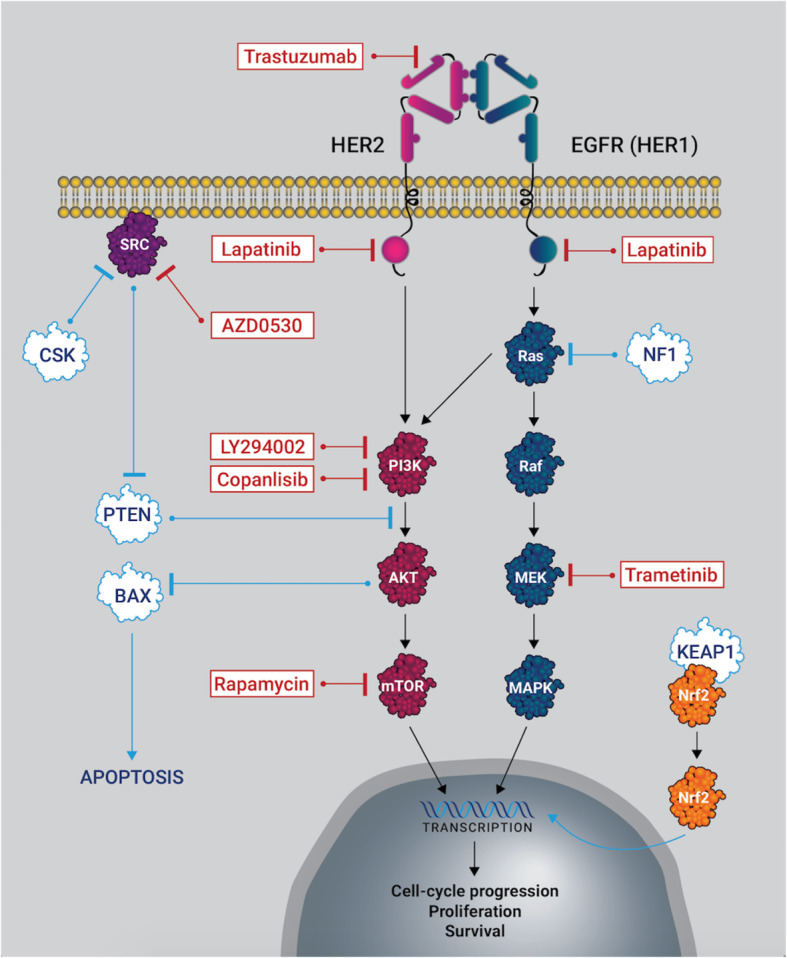
A schematic diagram showing potential HER2-related signaling pathways and action mechanisms of various inhibitors in HER2-amplified GC. Heterodimerization of HER2 with other HER family members (such as EGFR, HER3, HER4) result in tyrosine kinase activation with the subsequent signaling cascade, including members of MAPK and PI3K/AKT/mTOR pathways. As a result of these signaling pathways activation, different nuclear factors are recruited and modulate the transcription of different genes involved in cell-cycle progression, proliferation, and survival. Trastuzumab inhibits HER2 by targeting its extracellular domain, whereas lapatinib inhibits both HER2 and EGFR by inhibiting the intracellular tyrosine kinases. HER2 targeted therapy could be interrupted by re-activation MAPK and PI3K/AKT/mTOR pathways by compensatory activation of MET, IGF-RI, HER3 or loss of function mutations of tumor suppressor genes such as CSK, PTEN, NF1. In addition, drug resistance could be conferred by loss of function mutations of downstream genes such as KEAP1 and BAX by dysregulation of cellular antioxidants, xenobiotic detoxification enzymes and apoptosis, respectively. Lapatinib combining with lapatinib, SRC inhibitor AZD0530, PI3K inhibitor copanlisib, mTOR inhibitor rapamycin or MEK inhibitor trametinib could counteract the resistance at different level, respectively. A combinational treatment strategy with lapatinib, copanlisib and trametinib is demonstrated more effective for HER2 amplified GC with CSK, PTEN, NF1and KEAP1 mutations in this study

In this study, we also identified and validated other genes that may be involved in lapatinib resistance, such as NF1 and KEAP1 (Fig. S2 and Fig. S3). Interestingly, we found that lapatinib in combination with PI3K inhibitor copanlisib and MEK inhibitor trametinib could also overcome the resistance to lapatinib conferred by NF1 and KEAP1 knockout (Fig. S4D). Although the mechanism is not elucidated in GC, loss of NF1 has been associated with resistance to EGFR TKIs in lung adenocarcinomas and resistance to BRAF inhibitor in melanoma by increasing MAPK and/or PI3K signaling via negatively regulating Ras [44, 45]. Previous studies suggest that loss of KEAP1 function may lead to the nuclear translocation of Nrf2 and subsequent increased expression of cellular antioxidants and xenobiotic detoxification enzymes, which may be the major resistance mechanism of tumor cells against chemotherapeutic drugs [46, 47]. Coincidentally, Nrf2 activation caused by loss of KEAP1 could be blocked by PI3K inhibitor [48], which is consistent with our result of the combinational treatment on KEAP1 null GC cells. Taken together, PI3K and MAPK signaling may also play important roles in lapatinib resistance in the HER2-amplified GC patients bearing loss of function mutations of NF1 or KEAP1. Merging our finding with previous studies, we draw a schematic diagram showing potential HER2-related signaling pathways and action mechanisms of various inhibitors in HER2-amplified GC (Fig. 6). Additional studies would be helpful to elucidate the molecular mechanisms of the drug resistance induced by these gene mutations.

In summary, CRISPR library screening provides a valuable platform for gene function screening and novel drug target discovery. Our study has validated the approach and revealed the potential molecular mechanisms for the treatment of subsets of GC cases: loss-of-function mutation of CSK or PTEN causes resistance to lapatinib in HER2-amplified GC cells via hyperactivation of PI3K and MAPK pathways, which can be overcome by applying drug combination of lapatinib, PI3K and MAPK pathway inhibitors. The current study extends the understanding of lapatinib resistance in HER2-amplified GC, which would facilitate to develop alternative treatment strategy to increase efficacy of anti-HER2 treatment. To further confirm the efficacy of this new therapeutic strategy pre-clinically, a patient-derived xenograft (PDX) model-based study is expected in the near future.

## Conclusions

This study provides insights into understanding of the resistance mechanism of HER2 targeted therapy, that loss of function mutation of CSK and PTEN re-activate PI3K and MAPK pathways and substantially cause lapatinib resistance. And we developed the novel strategy targeting both PI3K and MAPK pathways that may ultimately overcome resistance or limited efficacy of lapatinib treatment for a subset of HER2-amplified GC cases.

## Supplementary Information

**Additional file 1: Fig. S1.** (A) CRISPR-Cas9 library sequencing data analysis workflow. (B) The distribution of sgRNA frequencies of the corresponding replicates of N87 and OE19 cells. (C) Comparison of different treatment conditions and biological replicates in the N87 and OE19 lapatinib screening. Each square in the lower left half of the matrix compares the normalized sgRNA read count between two biological samples. Sample labels for each axis are indicated on the diagonal. For the sample labels, N87 and OE19 are the cell line names; D14 means vehicle day 14 and L14 means lapatinib treated day 14. The last number 1 or 2 means the replicates. The Pearson correlation coefficient can be found in each square in the upper right half of the matrix. For example, the number 0.947 on the second row of top row indicates that the correlation coefficient between N87 vehicle day replicate 1 and replicate 2 is 0.947. Wilcoxon rank-sum test indicated the following, **significant difference from the control, *p* < 2.2e-16. **Fig. S2.** Validation of the screen that disruption of a few other candidate genes individually in GC cell lines causes resistance to lapatinib. (A) Cell viability curve of KEAP1, BAX, MED24 or TADA1 knockout OE19 cells treated with indicated doses of lapatinib, respectively. OE19 cells were transduced with lentivirus carrying sgRNAs targeting the indicated gene individually. The drug resistance of gene knockout and the control cells were measured by the relative percentage of cell viability. (B) Cell viability curve of KCTD5 or NF1 knockout OE19 cells treated with indicated doses of lapatinib, respectively. OE19 cells were transduced with lentivirus carrying sgRNAs targeting the indicated gene individually. The drug resistance of gene knockout and the control cells were examined by the relative percentage of cell viability. Unpaired Student’s *t* test indicated the following: *significant difference from the control, *p* < 0.05; **significant difference from the control, *p* < 0.01. **Fig. S3.** Protein interaction networks and the predicted partners were analyzed by STRING on the validated genes. CSK, PTEN, KEAP1, BAX, MED24, TADA1, KCTD5, NF1 (validated genes) and HER2 (ERBB2) were mapped by searching the STRING for Protein interaction networks (A) and the predicted partners analysis (B). In the resulting protein association network, proteins are presented as nodes which are connected by lines whose thickness represents the confidence level. (C) Heatmap of 139 DEGs between CSK null cells vs parental N87 cells (Fold change > 1.5. FDR < 0.1). (D) Heatmap of 997 DEGs between PTEN null cells vs parental OE19 cells (Fold change > 1.5. FDR < 0.1). **Fig. S4.** Pharmacological test with trastuzumab, LY294002 and combination of lapatinib, trametinib and/or copanlisib on lapatinib resistant GC cells. (A) Growth curve of test groups of N87 cells with 0.01 μM lapatinib in combination with indicated doses of trastuzumab for 6 days. (B) Growth curve of CSK and PTEN gene knockout OE19 cell lines treated with 0.05 μM lapatinib in combination with different doses of PI3K inhibitor LY294002 for 6 days. OE19 cells transduced with non-targeting sgRNA as control. (C) Pharmacological inhibition of PI3K, MAPK signaling pathway re-sensitizes CSK or PTEN null GC cells to lapatinib. Inhibition effect of 0.01 μM lapatinib alone or in combination with 0.1 μM trametinib or /and 0.1 μM copanlisib for 6 days. N87 cells transduced with CSK targeting sgRNAs or PTEN targeting sgRNAs were used for the test. N87 cells transduced with non-targeting sgRNA as control. (D) Pharmacological inhibition of PI3K, MAPK signaling pathway re-sensitizes NF1 or KEAP1 null GC cells to lapatinib. Inhibition effect of 0.05 μM lapatinib alone or in combination with 0.1 μM trametinib or /and 0.1 μM copanlisib for 6 days. OE19 cells transduced with NF1 targeting sgRNAs or KEAP1 targeting sgRNAs were used for the test. OE19 cells transduced with non-targeting sgRNA as control. Unpaired Student’s *t* test indicated the following: *significant difference from the control, *p* < 0.05; **significant difference from the control, *p* < 0.01. **Fig. S5.** Lapatinib response curve of N87-WT, N87-CSK^−^/^−^ or N87-PTEN^−^/^−^ xenograft tumors. Lapatinib at concentration of 50 mg/kg, 100 mg/kg and 200 mg/kg were given to xenografts bearing N87-WT (A), N87-CSK^−^/^−^ (B) or N87-PTEN^−^/^−^ (C) tumor by oral gavage. Tumor sizes were measured twice a week.

**Additional file 2.**

## Data Availability

All deep sequencing data are available at GEO (series accession number GSE148668) [https://www.ncbi.nlm.nih.gov/geo/query/acc.cgi?acc=GSE148668].

## Abbreviations

GC: Gastric cancer
HER2: Human epidermal growth factor receptor 2
MAPK: Ras/Raf/Mitogen-activated protein kinase
PI3K: Phosphatidylinositol 3 kinase
mTOR: Mammalian target of rapamycin
shRNA: Short hairpin RNA
IACUC: Institutional Animal Care and Use Committee
NSG: NOD/SCID/IL-2γ-receptor null
MAGeCK: Genome-wide CRISPR-Cas9 Knockout
RRA: Robust Rank Aggregation
MLE: MAGeCK Maximum Likelihood Estimation
WES: Whole-exome Sequencing
BWA: Burrows-Wheeler Aligner
GATK: Genome Analysis Toolkit
SNVs: Single nucleotide variants
CNVs: Copy number variants
MOI: Multiplicity of infection
sgRNAs: Single-guide RNAs
STRING: Search Tool for the Retrieval of Interacting Genes
DEGs: Differentially expressed genes
KEGG: Kyoto Encyclopedia of Genes and Genomes
RTKs: Receptor tyrosine kinases
PDX: Patient-derived xenograft
