# Supplementary material for: A novel treatment strategy for lapatinib resistance in a subset of HER2-amplified gastric cancer
Source: BMC Cancer. 2021 Aug 16;21:923. doi: 10.1186/s12885-021-08283-9 (PMC8366014; doi:10.1186/s12885-021-08283-9)
Supplement: Supplementary file 1 — Additional file 1: Fig. S1. (A) CRISPR-Cas9 library sequencing data analysis workflow. (B) The distribution of sgRNA frequencies of the corresponding replicates of N87 and OE19 cells. (C) Comparison of different treatment conditions and biological replicates in the N87 and OE19 lapatinib screening. Each square in the lower left half of the matrix compares the normalized sgRNA read count between two biological samples. Sample labels for each axis are indicated on the diagonal. For the sample labels, N87 and OE19 are the cell line names; D14 means vehicle day 14 and L14 means lapatinib treated day 14. The last number 1 or 2 means the replicates. The Pearson correlation coefficient can be found in each square in the upper right half of the matrix. For example, the number 0.947 on the second row of top row indicates that the correlation coefficient between N87 vehicle day replicate 1 and replicate 2 is 0.947. Wilcoxon rank-sum test indicated the following, **significant difference from the control, p < 2.2e-16. Fig. S2. Validation of the screen that disruption of a few other candidate genes individually in GC cell lines causes resistance to lapatinib. (A) Cell viability curve of KEAP1, BAX, MED24 or TADA1 knockout OE19 cells treated with indicated doses of lapatinib, respectively. OE19 cells were transduced with lentivirus carrying sgRNAs targeting the indicated gene individually. The drug resistance of gene knockout and the control cells were measured by the relative percentage of cell viability. (B) Cell viability curve of KCTD5 or NF1 knockout OE19 cells treated with indicated doses of lapatinib, respectively. OE19 cells were transduced with lentivirus carrying sgRNAs targeting the indicated gene individually. The drug resistance of gene knockout and the control cells were examined by the relative percentage of cell viability. Unpaired Student’s t test indicated the following: *significant difference from the control, p < 0.05; **significant difference from the contr [file 12885_2021_8283_MOESM1_ESM.pptx]

## Slide 1
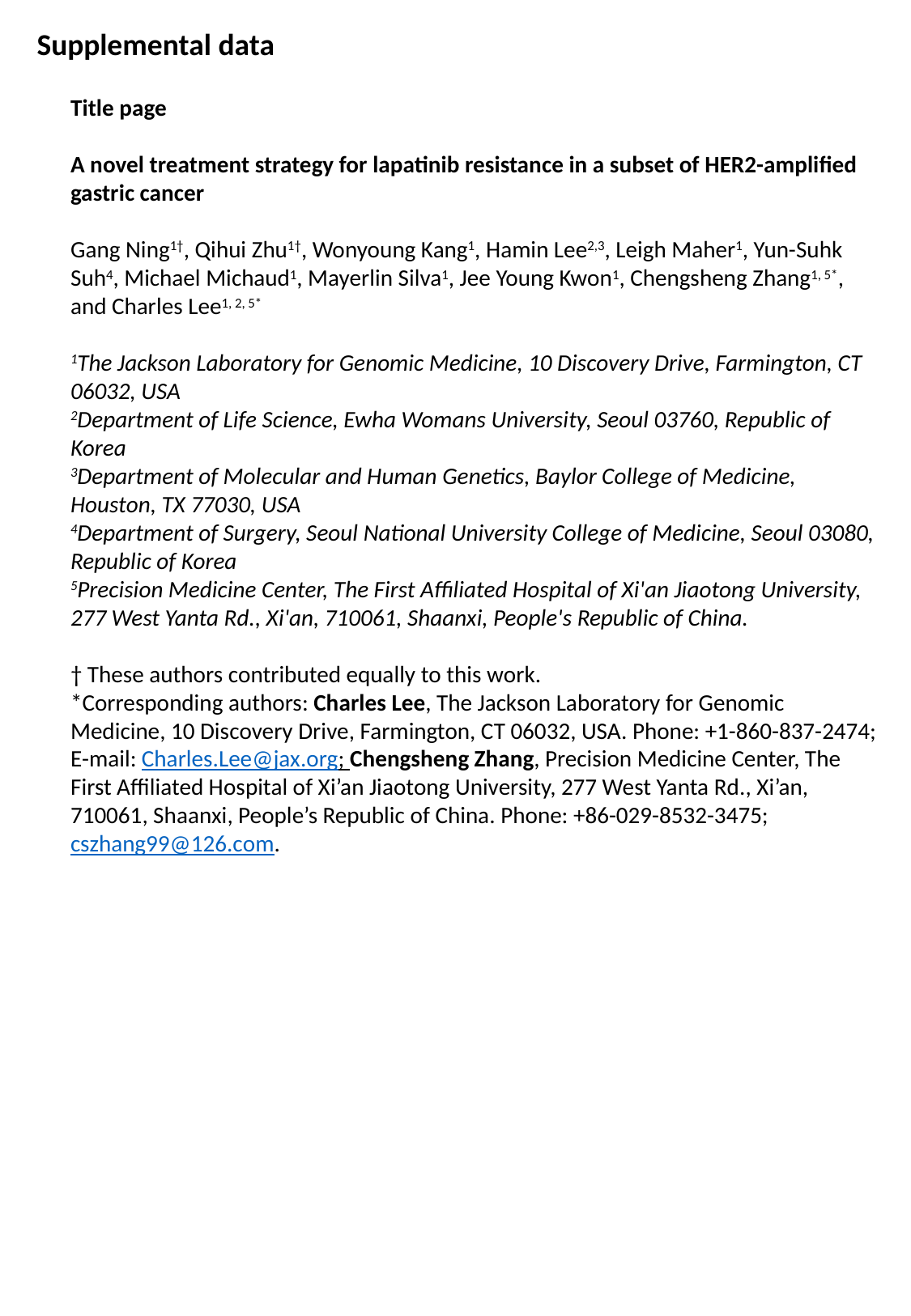

Supplemental data
Title page
A novel treatment strategy for lapatinib resistance in a subset of HER2-amplified gastric cancer
Gang Ning1†, Qihui Zhu1†, Wonyoung Kang1, Hamin Lee2,3, Leigh Maher1, Yun-Suhk Suh4, Michael Michaud1, Mayerlin Silva1, Jee Young Kwon1, Chengsheng Zhang1, 5*, and Charles Lee1, 2, 5*
1The Jackson Laboratory for Genomic Medicine, 10 Discovery Drive, Farmington, CT 06032, USA
2Department of Life Science, Ewha Womans University, Seoul 03760, Republic of Korea
3Department of Molecular and Human Genetics, Baylor College of Medicine, Houston, TX 77030, USA
4Department of Surgery, Seoul National University College of Medicine, Seoul 03080, Republic of Korea
5Precision Medicine Center, The First Affiliated Hospital of Xi'an Jiaotong University, 277 West Yanta Rd., Xi'an, 710061, Shaanxi, People's Republic of China.
† These authors contributed equally to this work.
*Corresponding authors: Charles Lee, The Jackson Laboratory for Genomic Medicine, 10 Discovery Drive, Farmington, CT 06032, USA. Phone: +1-860-837-2474; E-mail: Charles.Lee@jax.org; Chengsheng Zhang, Precision Medicine Center, The First Affiliated Hospital of Xi’an Jiaotong University, 277 West Yanta Rd., Xi’an, 710061, Shaanxi, People’s Republic of China. Phone: +86-029-8532-3475; cszhang99@126.com.

## Slide 2
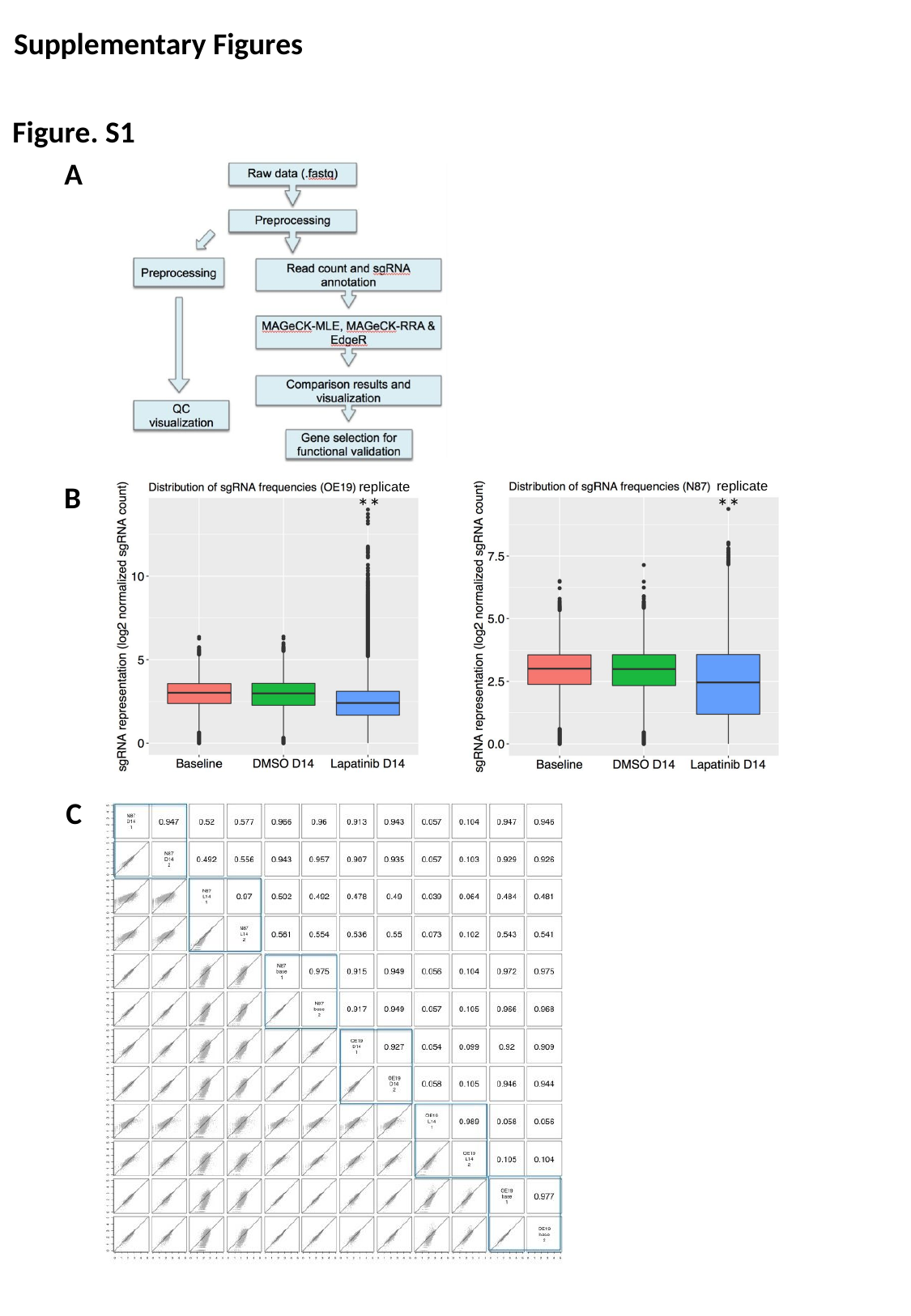

Supplementary Figures
Figure. S1
A
replicate
B
replicate
**
**
C

## Slide 3
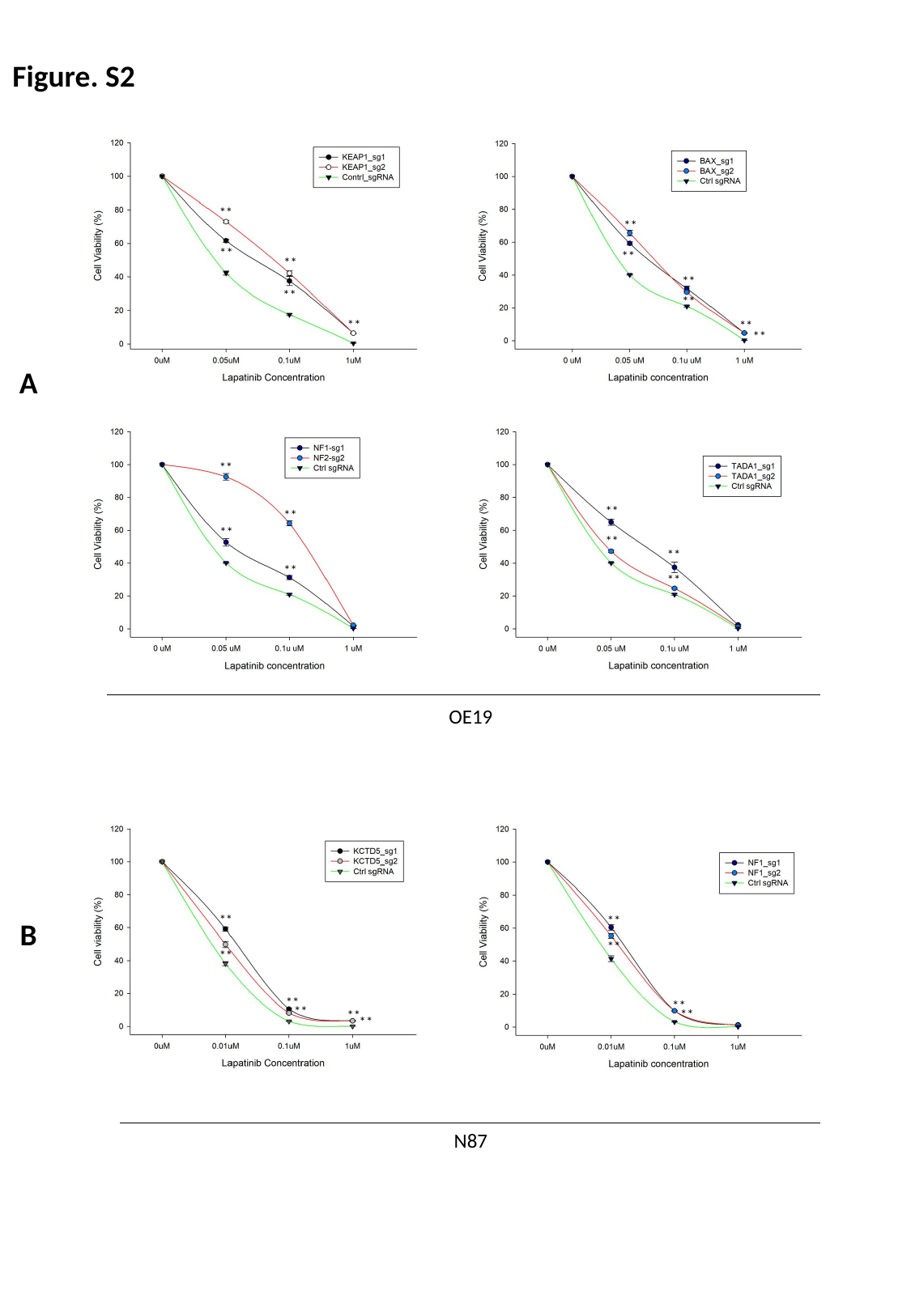

Figure. S2
**
**
**
**
**
**
**
**
**
**
**
A
**
**
**
**
**
**
**
**
OE19
**
**
B
**
**
**
**
**
**
**
**
N87

## Slide 4
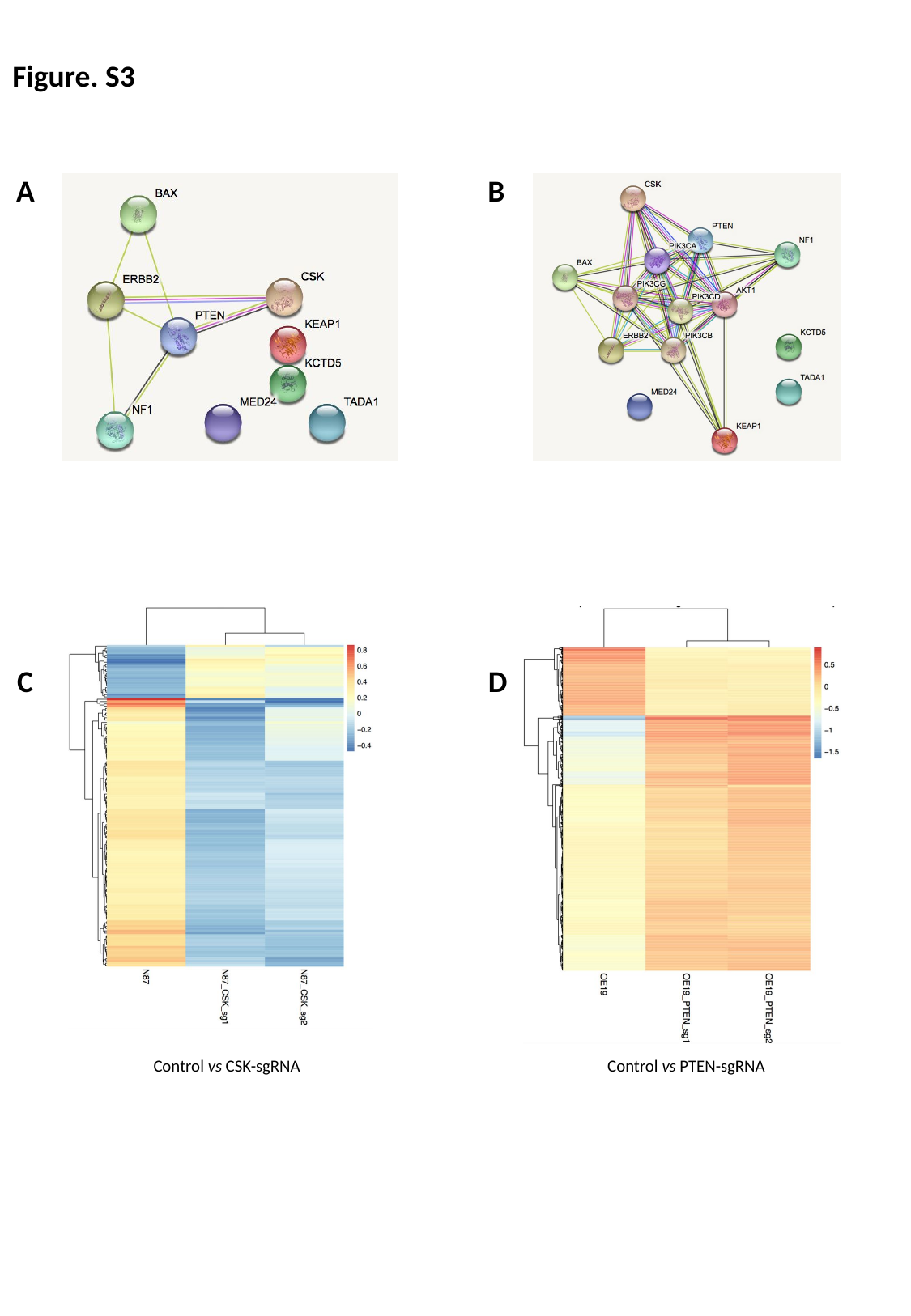

Figure. S3
A
B
C
D
Control vs CSK-sgRNA
Control vs PTEN-sgRNA

## Slide 5
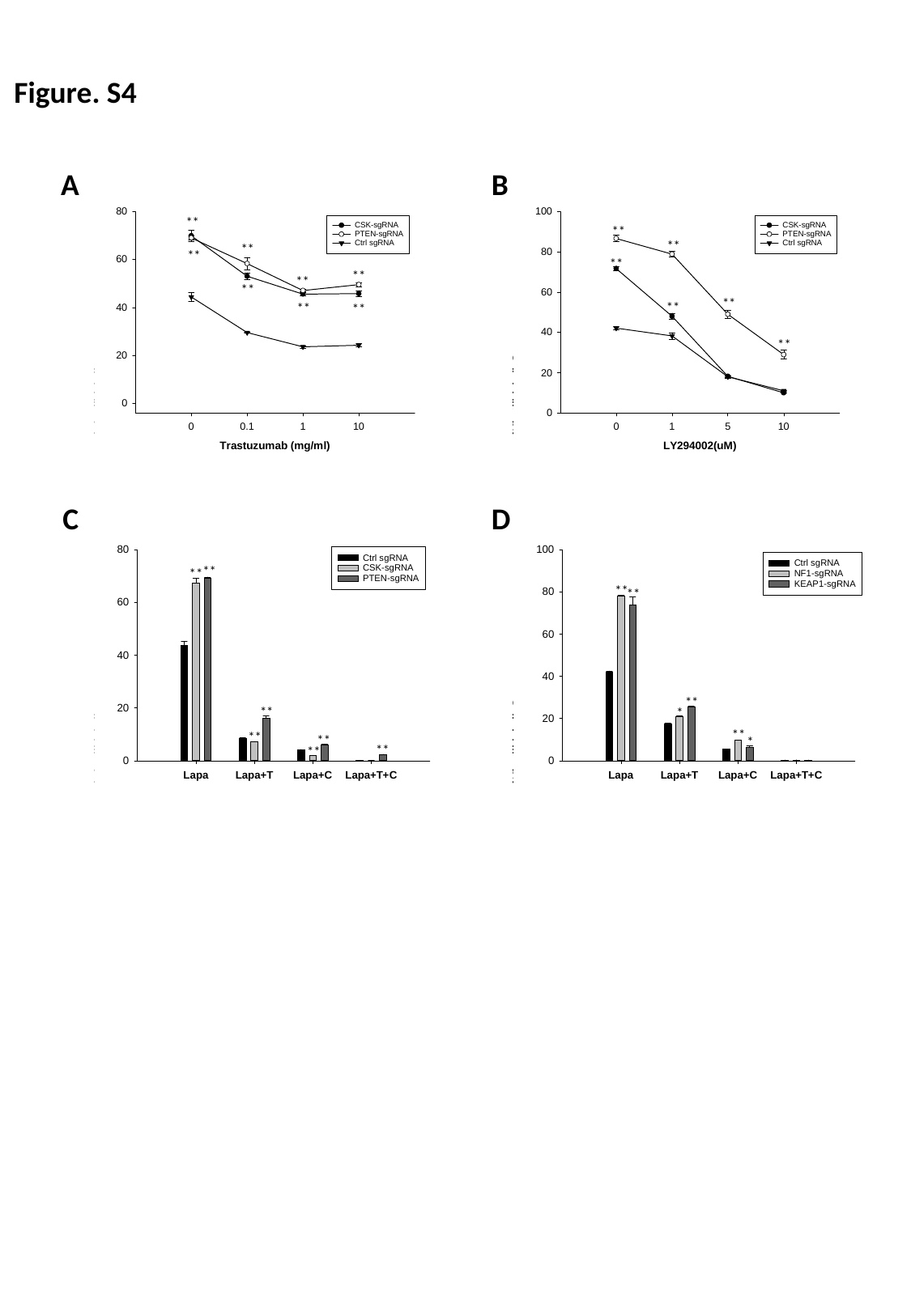

Figure. S4
A
B
**
**
**
**
**
**
**
**
**
**
**
**
**
**
C
D
**
**
**
**
**
**
*
**
**
**
*
**
**

## Slide 6
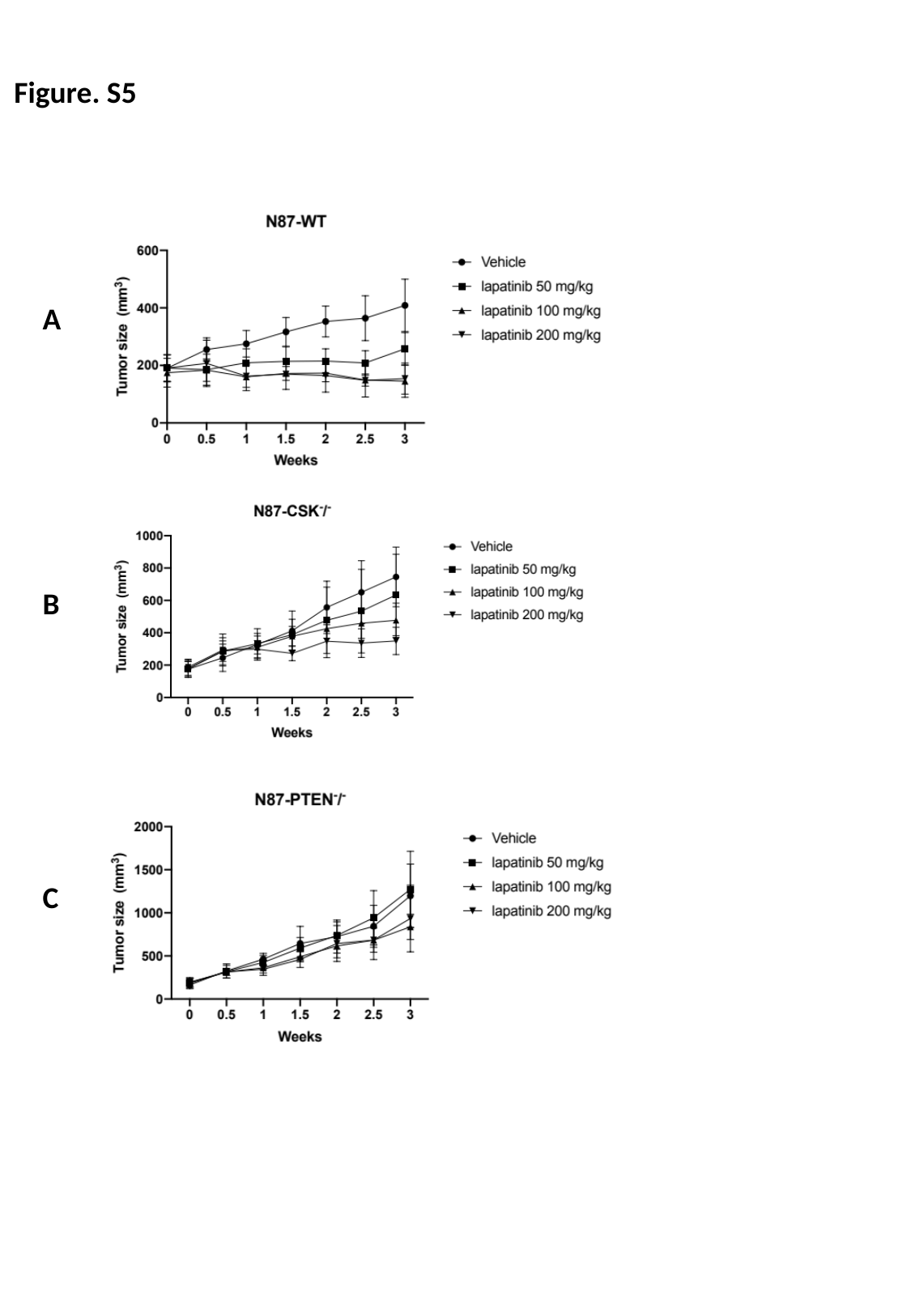

Figure. S5
A
B
C

## Slide 7
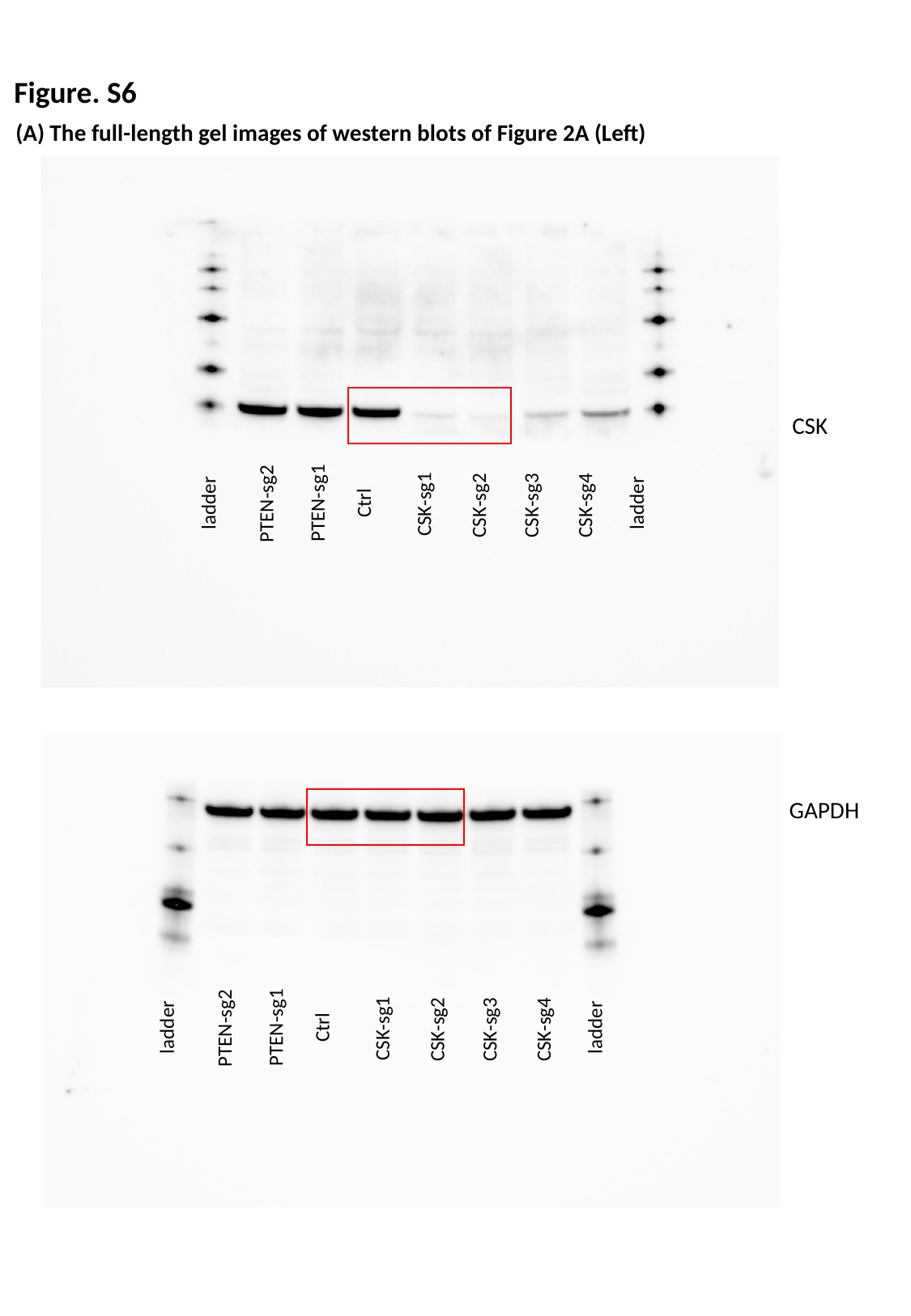

Figure. S6
(A) The full-length gel images of western blots of Figure 2A (Left)
CSK
ladder
ladder
Ctrl
PTEN-sg1
PTEN-sg2
CSK-sg1
CSK-sg2
CSK-sg3
CSK-sg4
GAPDH
ladder
ladder
Ctrl
PTEN-sg1
PTEN-sg2
CSK-sg1
CSK-sg2
CSK-sg3
CSK-sg4

## Slide 8
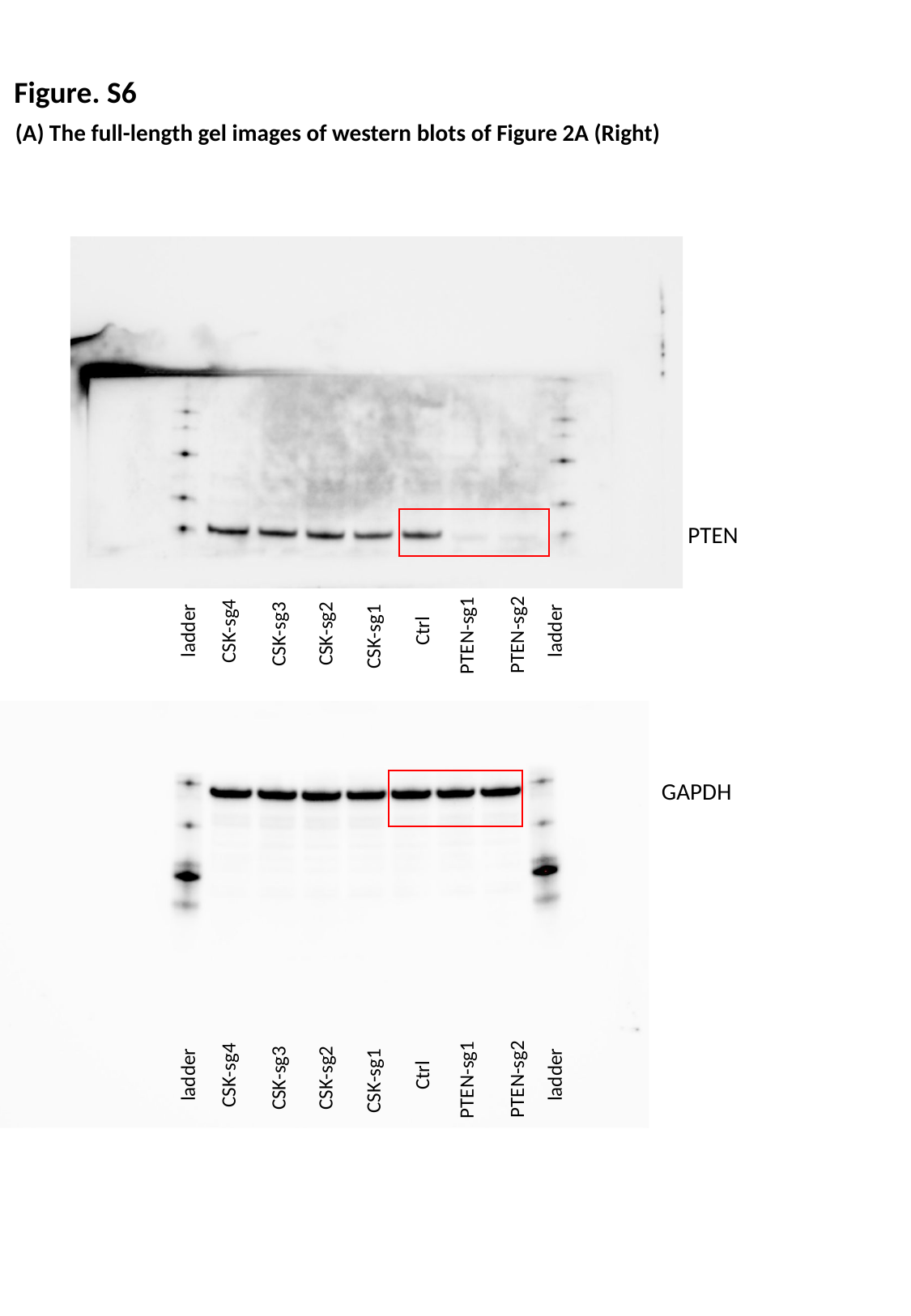

Figure. S6
(A) The full-length gel images of western blots of Figure 2A (Right)
PTEN
CSK-sg3
ladder
ladder
Ctrl
CSK-sg4
CSK-sg1
CSK-sg2
PTEN-sg2
PTEN-sg1
GAPDH
CSK-sg3
ladder
ladder
Ctrl
CSK-sg4
CSK-sg1
CSK-sg2
PTEN-sg2
PTEN-sg1

## Slide 9
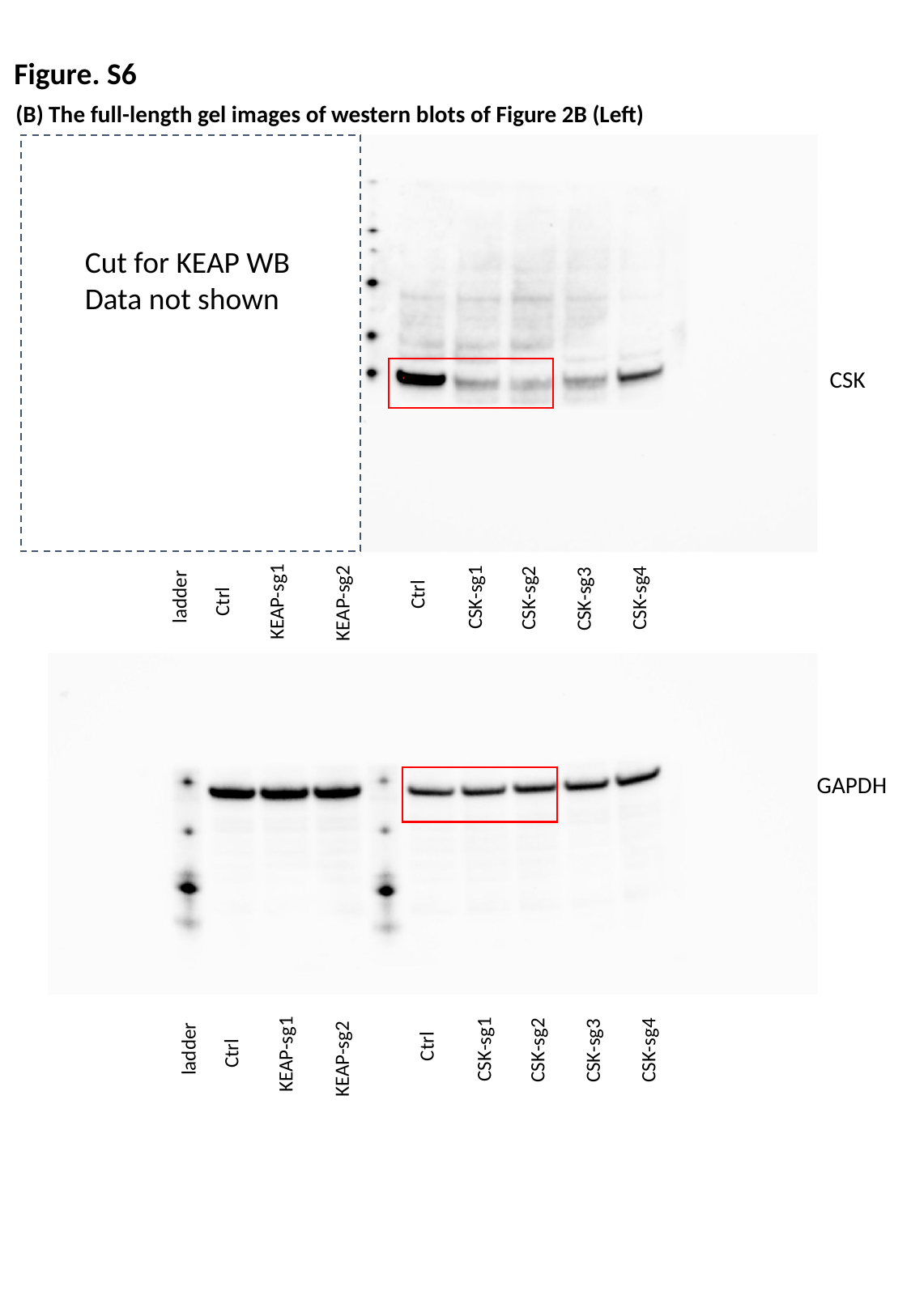

Figure. S6
(B) The full-length gel images of western blots of Figure 2B (Left)
Cut for KEAP WB
Data not shown
CSK
Ctrl
ladder
CSK-sg1
CSK-sg4
CSK-sg2
CSK-sg3
Ctrl
KEAP-sg1
KEAP-sg2
GAPDH
Ctrl
ladder
CSK-sg1
CSK-sg4
CSK-sg2
CSK-sg3
Ctrl
KEAP-sg1
KEAP-sg2

## Slide 10
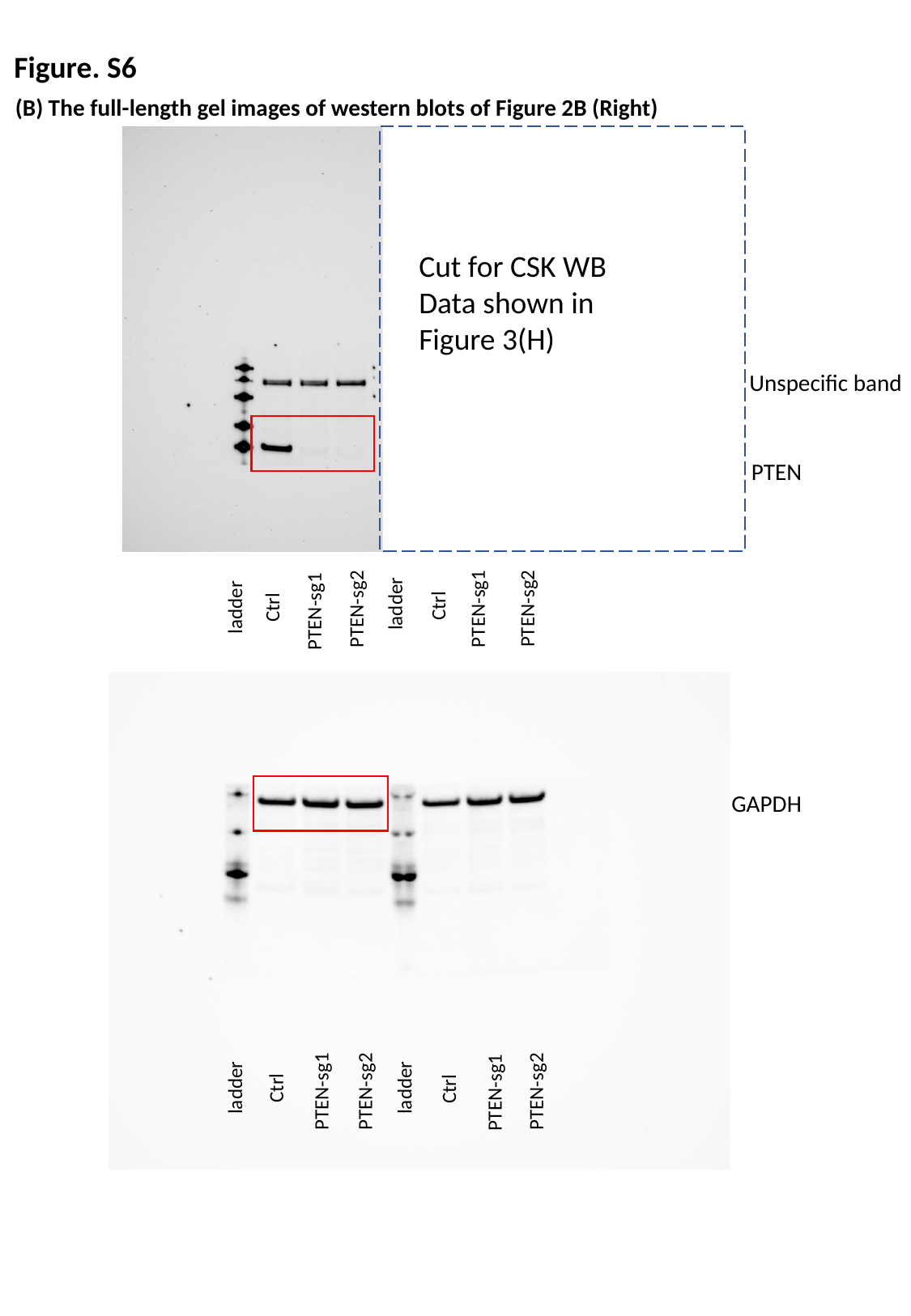

Figure. S6
(B) The full-length gel images of western blots of Figure 2B (Right)
Cut for CSK WB
Data shown in
Figure 3(H)
Unspecific band
PTEN
ladder
Ctrl
ladder
Ctrl
PTEN-sg2
PTEN-sg1
PTEN-sg2
PTEN-sg1
GAPDH
ladder
ladder
Ctrl
Ctrl
PTEN-sg1
PTEN-sg2
PTEN-sg2
PTEN-sg1

## Slide 11
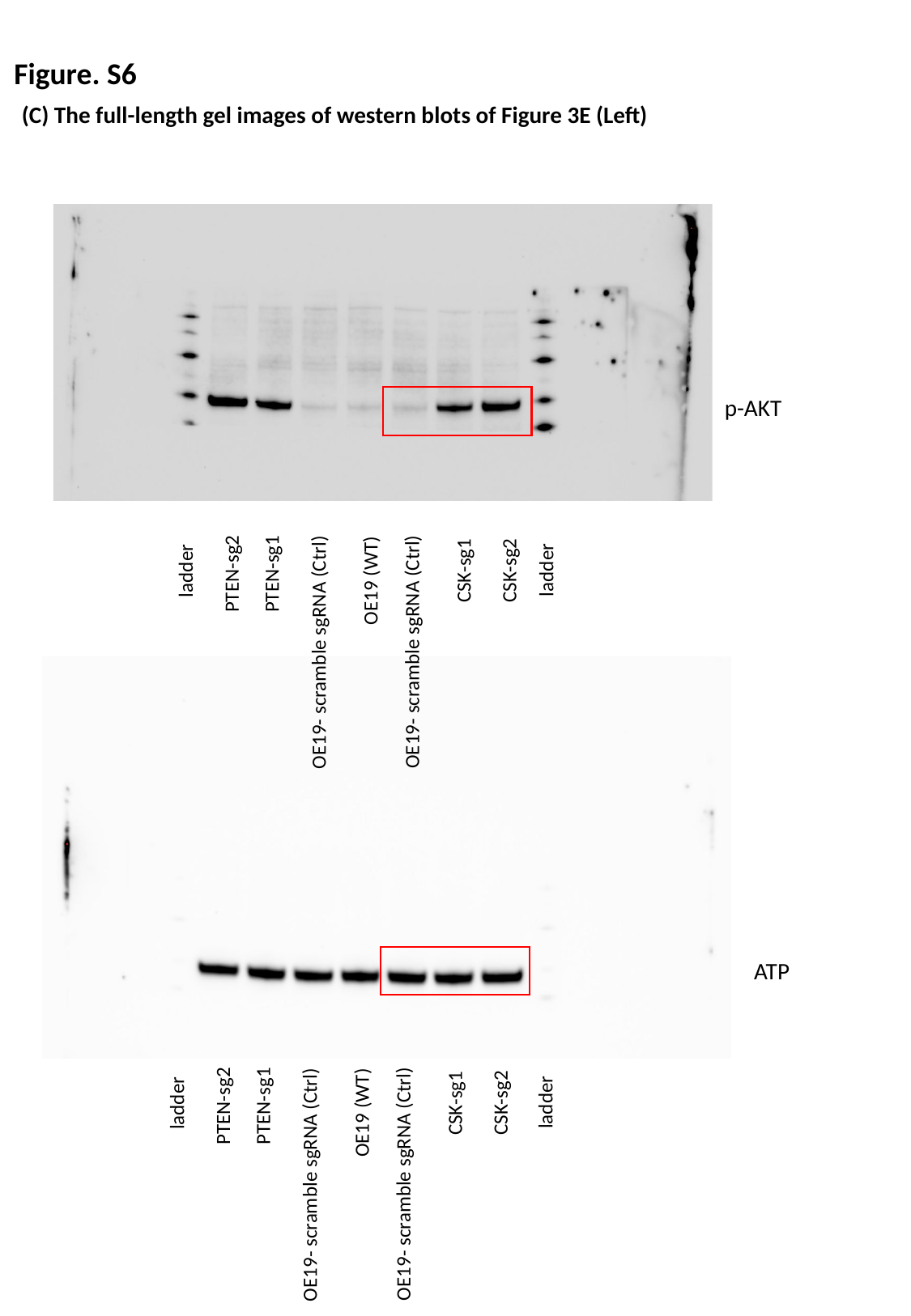

Figure. S6
(C) The full-length gel images of western blots of Figure 3E (Left)
p-AKT
ladder
CSK-sg2
CSK-sg1
ladder
PTEN-sg2
PTEN-sg1
OE19 (WT)
OE19- scramble sgRNA (Ctrl)
OE19- scramble sgRNA (Ctrl)
ATP
ladder
CSK-sg2
CSK-sg1
ladder
PTEN-sg2
PTEN-sg1
OE19 (WT)
OE19- scramble sgRNA (Ctrl)
OE19- scramble sgRNA (Ctrl)

## Slide 12
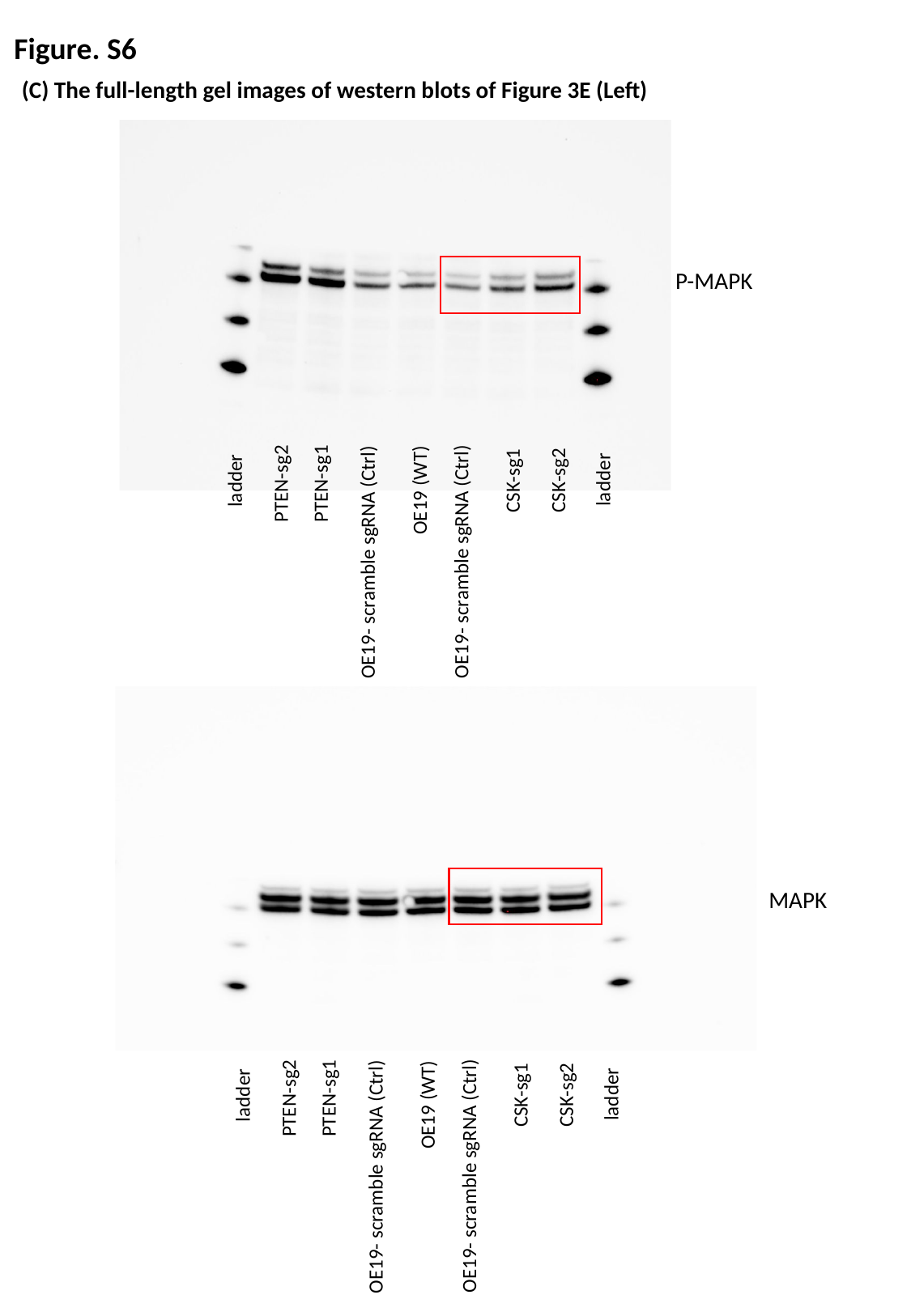

Figure. S6
(C) The full-length gel images of western blots of Figure 3E (Left)
P-MAPK
ladder
CSK-sg2
CSK-sg1
ladder
PTEN-sg2
PTEN-sg1
OE19 (WT)
OE19- scramble sgRNA (Ctrl)
OE19- scramble sgRNA (Ctrl)
MAPK
ladder
CSK-sg2
CSK-sg1
ladder
PTEN-sg2
PTEN-sg1
OE19 (WT)
OE19- scramble sgRNA (Ctrl)
OE19- scramble sgRNA (Ctrl)

## Slide 13
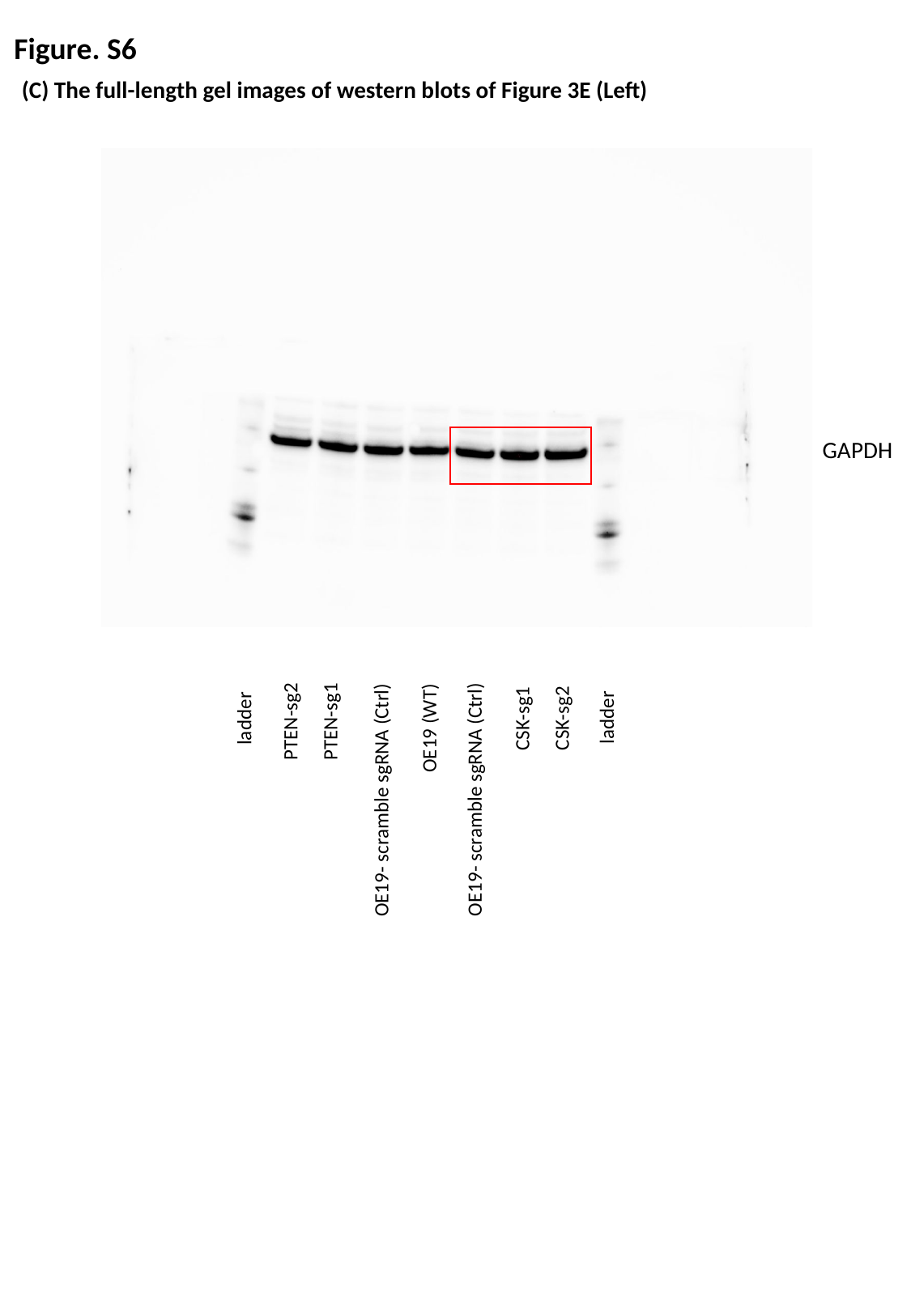

Figure. S6
(C) The full-length gel images of western blots of Figure 3E (Left)
GAPDH
ladder
CSK-sg2
CSK-sg1
ladder
PTEN-sg2
PTEN-sg1
OE19 (WT)
OE19- scramble sgRNA (Ctrl)
OE19- scramble sgRNA (Ctrl)

## Slide 14
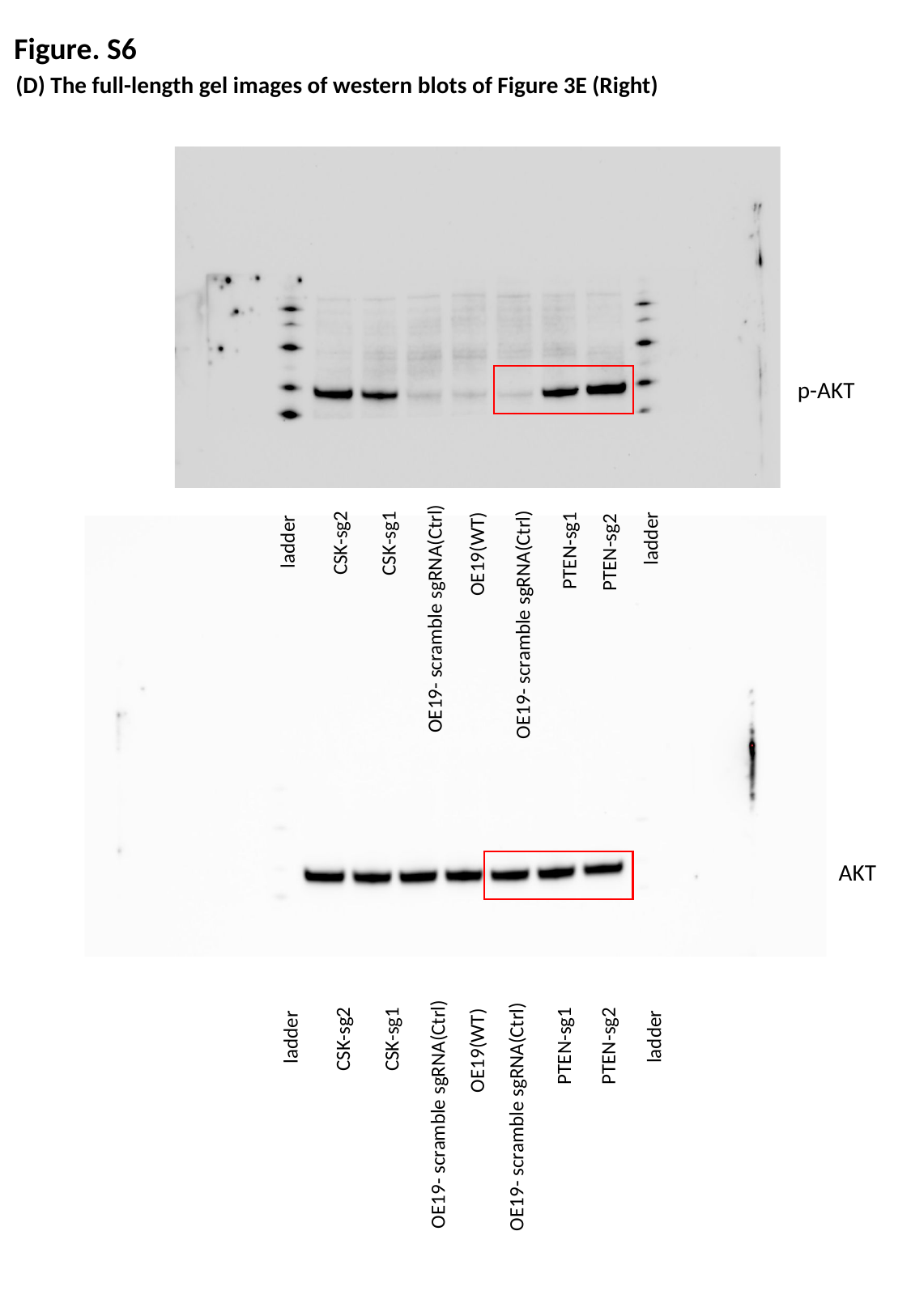

Figure. S6
(D) The full-length gel images of western blots of Figure 3E (Right)
p-AKT
ladder
OE19(WT)
ladder
CSK-sg2
CSK-sg1
PTEN-sg1
PTEN-sg2
OE19- scramble sgRNA(Ctrl)
OE19- scramble sgRNA(Ctrl)
AKT
ladder
ladder
OE19(WT)
CSK-sg2
CSK-sg1
PTEN-sg2
PTEN-sg1
OE19- scramble sgRNA(Ctrl)
OE19- scramble sgRNA(Ctrl)

## Slide 15
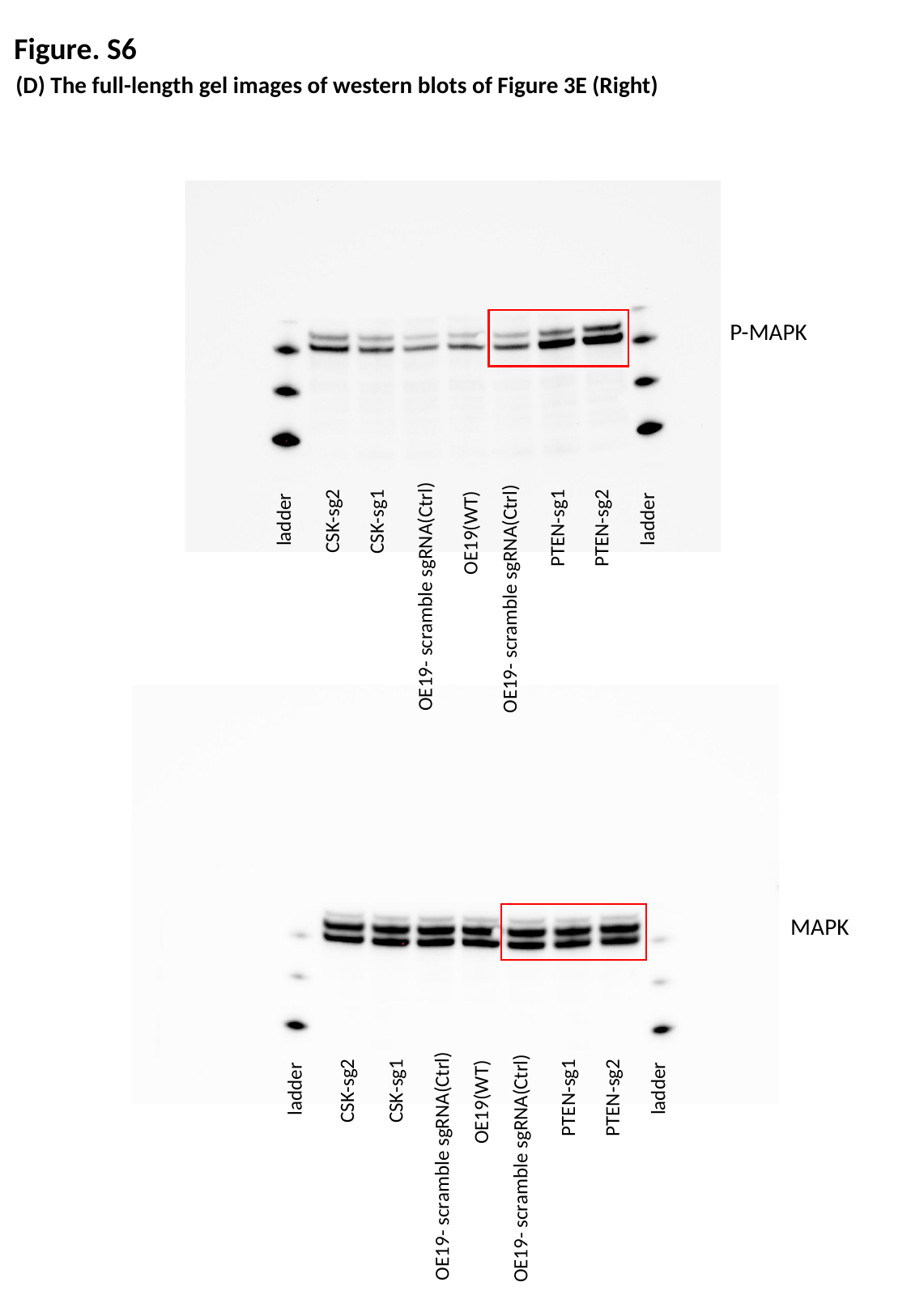

Figure. S6
(D) The full-length gel images of western blots of Figure 3E (Right)
P-MAPK
ladder
ladder
OE19(WT)
CSK-sg2
CSK-sg1
PTEN-sg2
PTEN-sg1
OE19- scramble sgRNA(Ctrl)
OE19- scramble sgRNA(Ctrl)
MAPK
ladder
ladder
OE19(WT)
CSK-sg2
CSK-sg1
PTEN-sg2
PTEN-sg1
OE19- scramble sgRNA(Ctrl)
OE19- scramble sgRNA(Ctrl)

## Slide 16
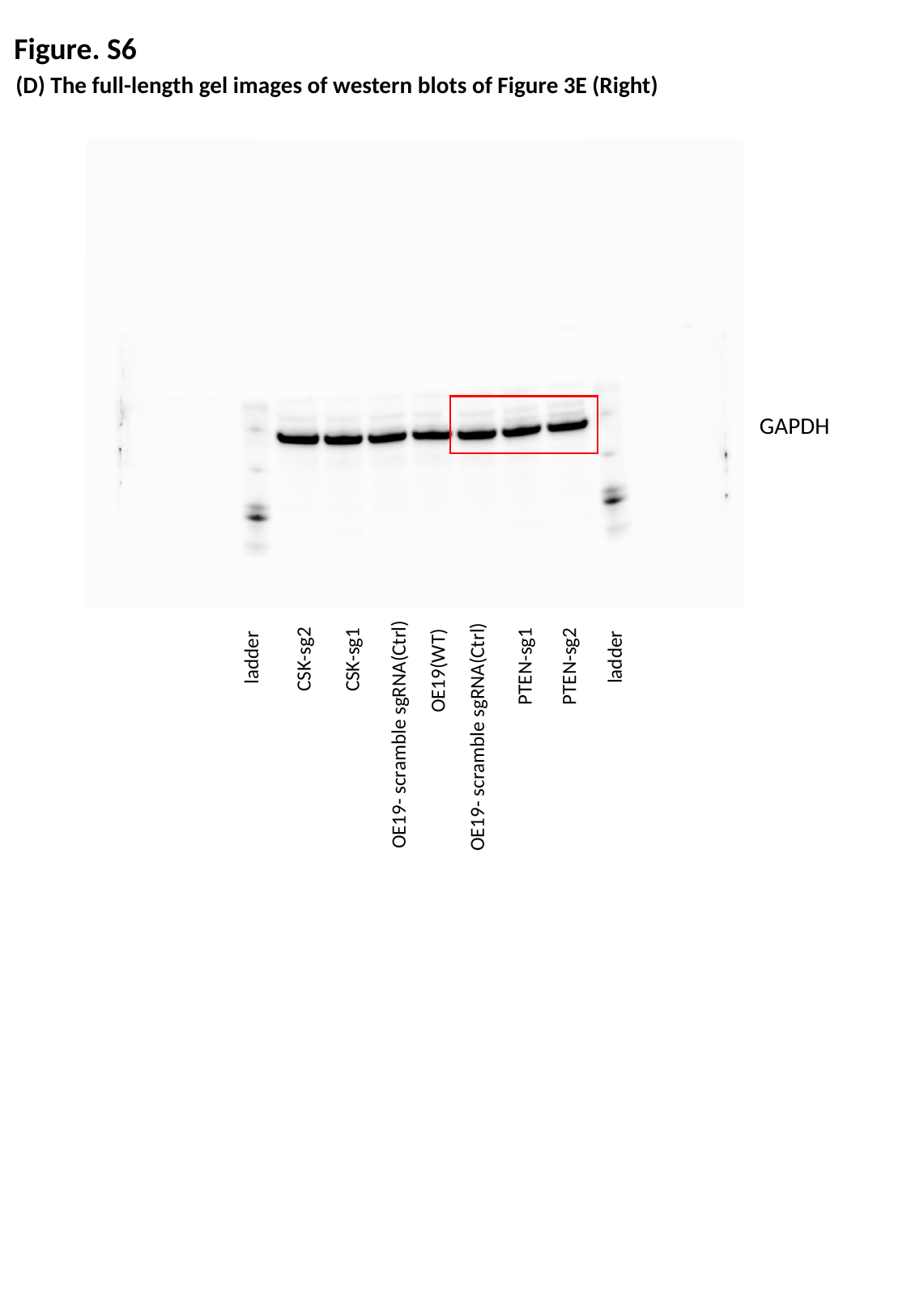

Figure. S6
(D) The full-length gel images of western blots of Figure 3E (Right)
GAPDH
ladder
ladder
OE19(WT)
CSK-sg2
CSK-sg1
PTEN-sg2
PTEN-sg1
OE19- scramble sgRNA(Ctrl)
OE19- scramble sgRNA(Ctrl)

## Slide 17
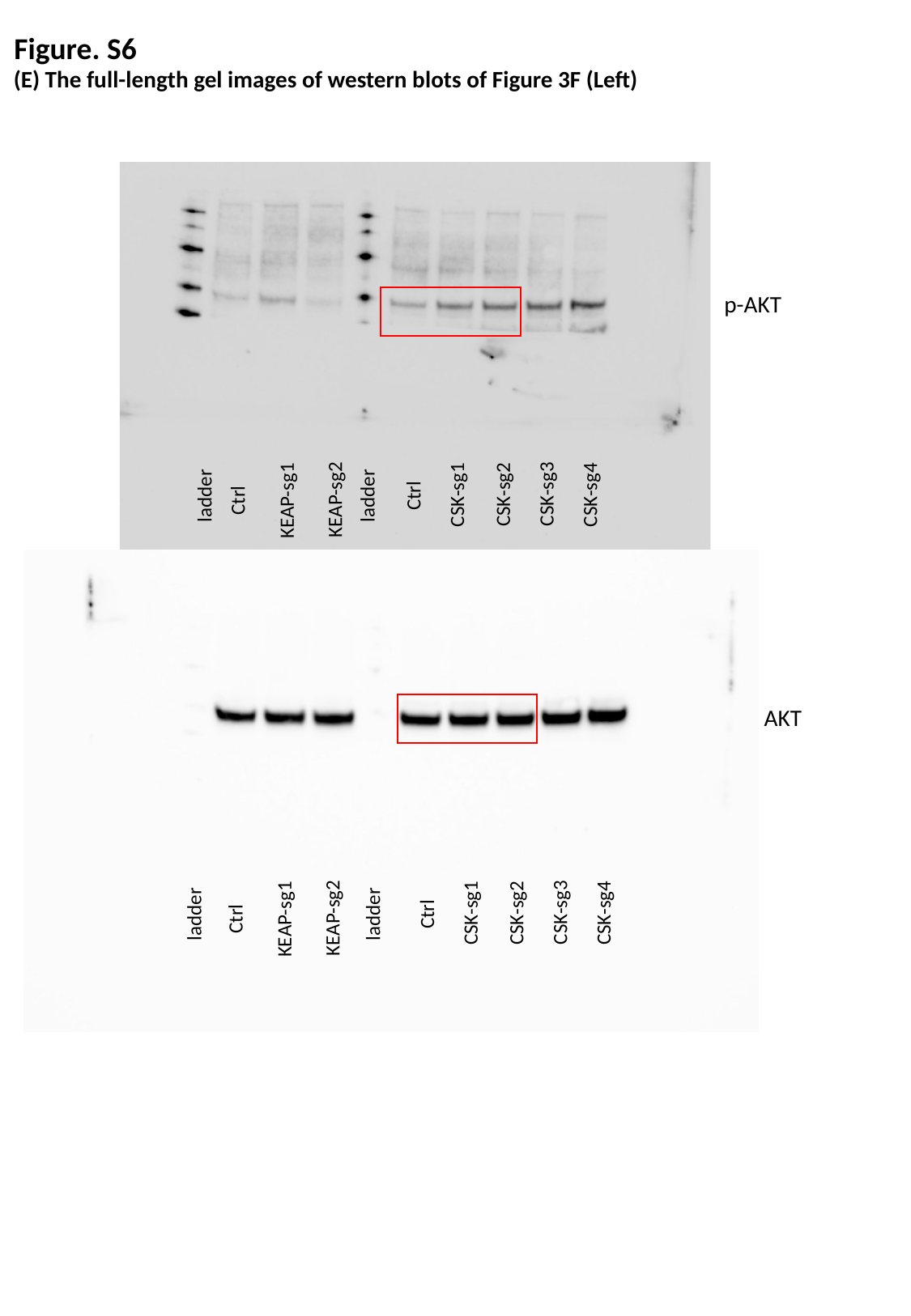

Figure. S6
(E) The full-length gel images of western blots of Figure 3F (Left)
p-AKT
CSK-sg3
CSK-sg2
CSK-sg1
CSK-sg4
Ctrl
ladder
ladder
KEAP-sg2
Ctrl
KEAP-sg1
AKT
CSK-sg3
CSK-sg2
CSK-sg1
CSK-sg4
Ctrl
ladder
ladder
KEAP-sg2
Ctrl
KEAP-sg1

## Slide 18
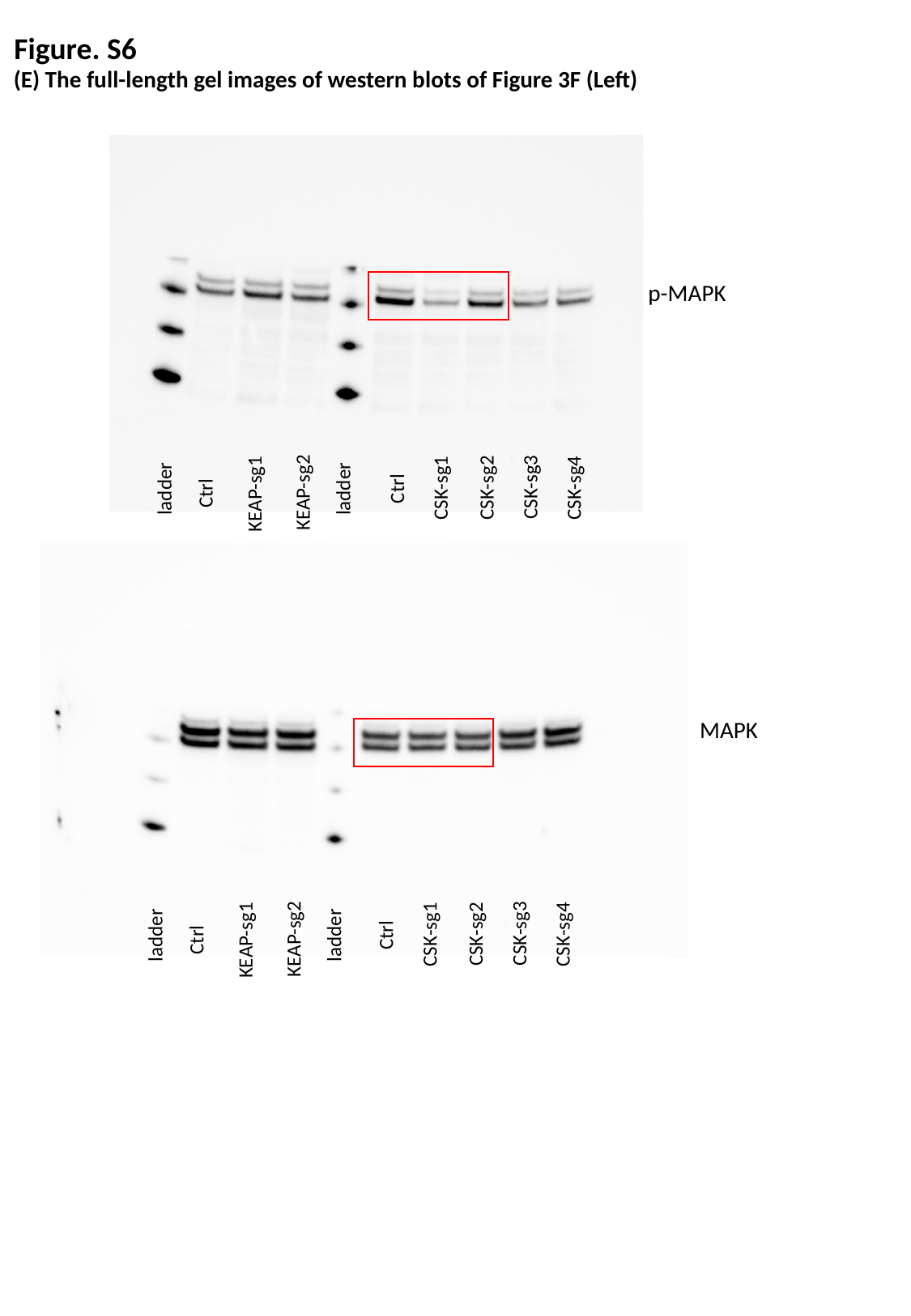

Figure. S6
(E) The full-length gel images of western blots of Figure 3F (Left)
p-MAPK
CSK-sg3
CSK-sg2
CSK-sg1
CSK-sg4
Ctrl
ladder
ladder
KEAP-sg2
Ctrl
KEAP-sg1
MAPK
CSK-sg3
CSK-sg2
CSK-sg1
CSK-sg4
Ctrl
ladder
ladder
KEAP-sg2
Ctrl
KEAP-sg1

## Slide 19
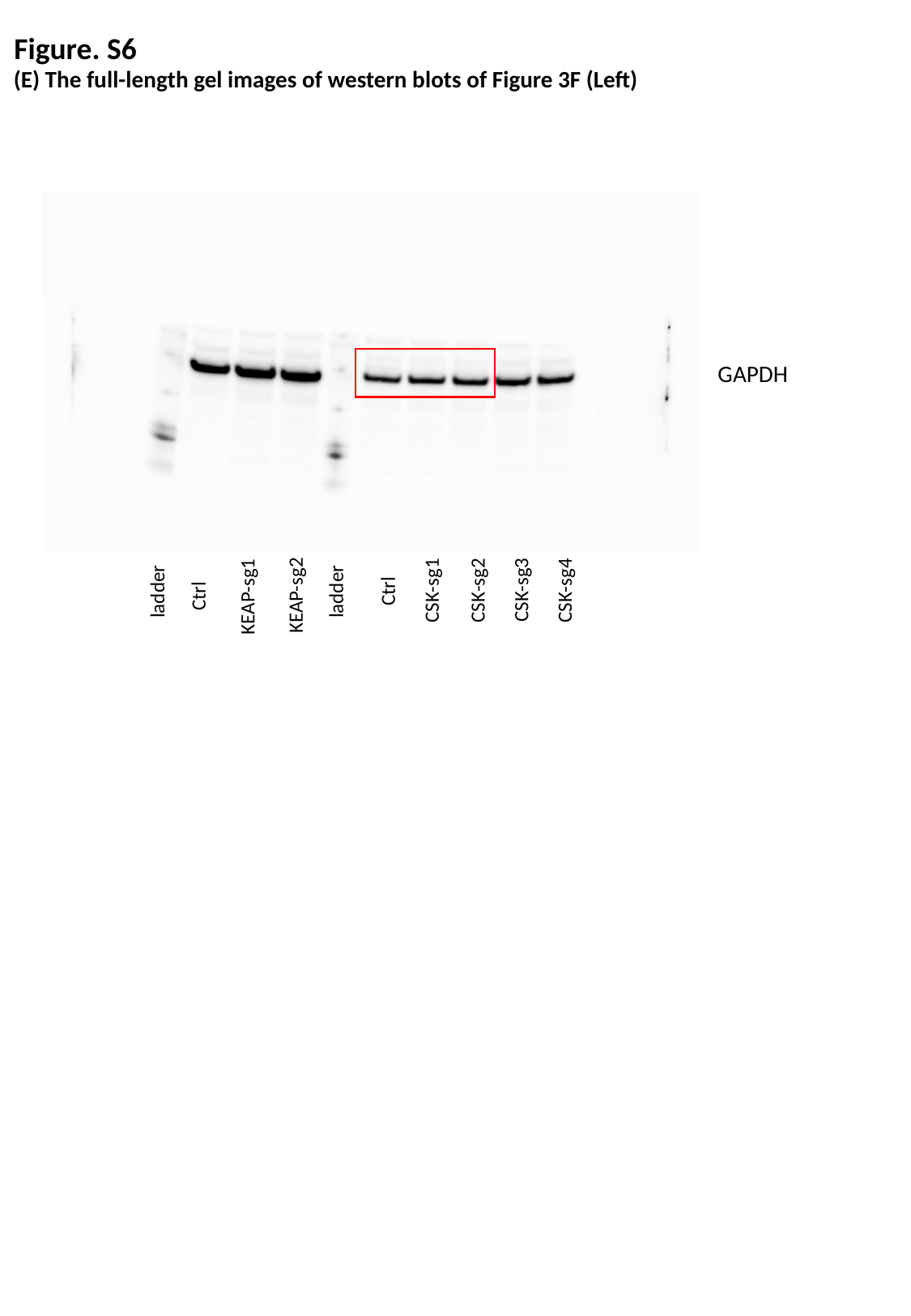

Figure. S6
(E) The full-length gel images of western blots of Figure 3F (Left)
GAPDH
CSK-sg3
CSK-sg2
CSK-sg1
CSK-sg4
Ctrl
ladder
ladder
KEAP-sg2
Ctrl
KEAP-sg1

## Slide 20
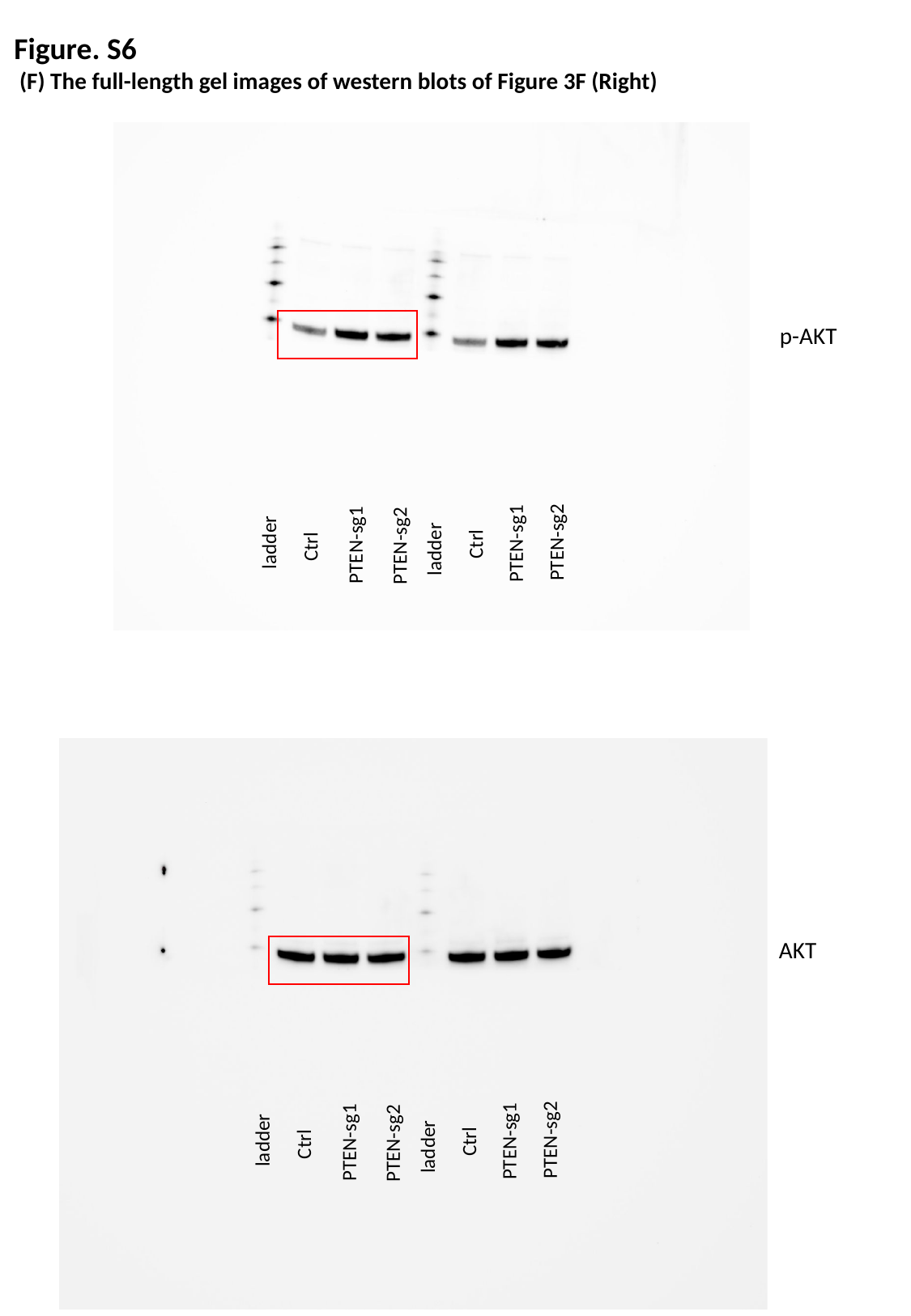

Figure. S6
(F) The full-length gel images of western blots of Figure 3F (Right)
p-AKT
PTEN-sg2
ladder
PTEN-sg1
Ctrl
PTEN-sg1
PTEN-sg2
Ctrl
ladder
AKT
PTEN-sg2
ladder
PTEN-sg1
Ctrl
PTEN-sg1
PTEN-sg2
Ctrl
ladder

## Slide 21
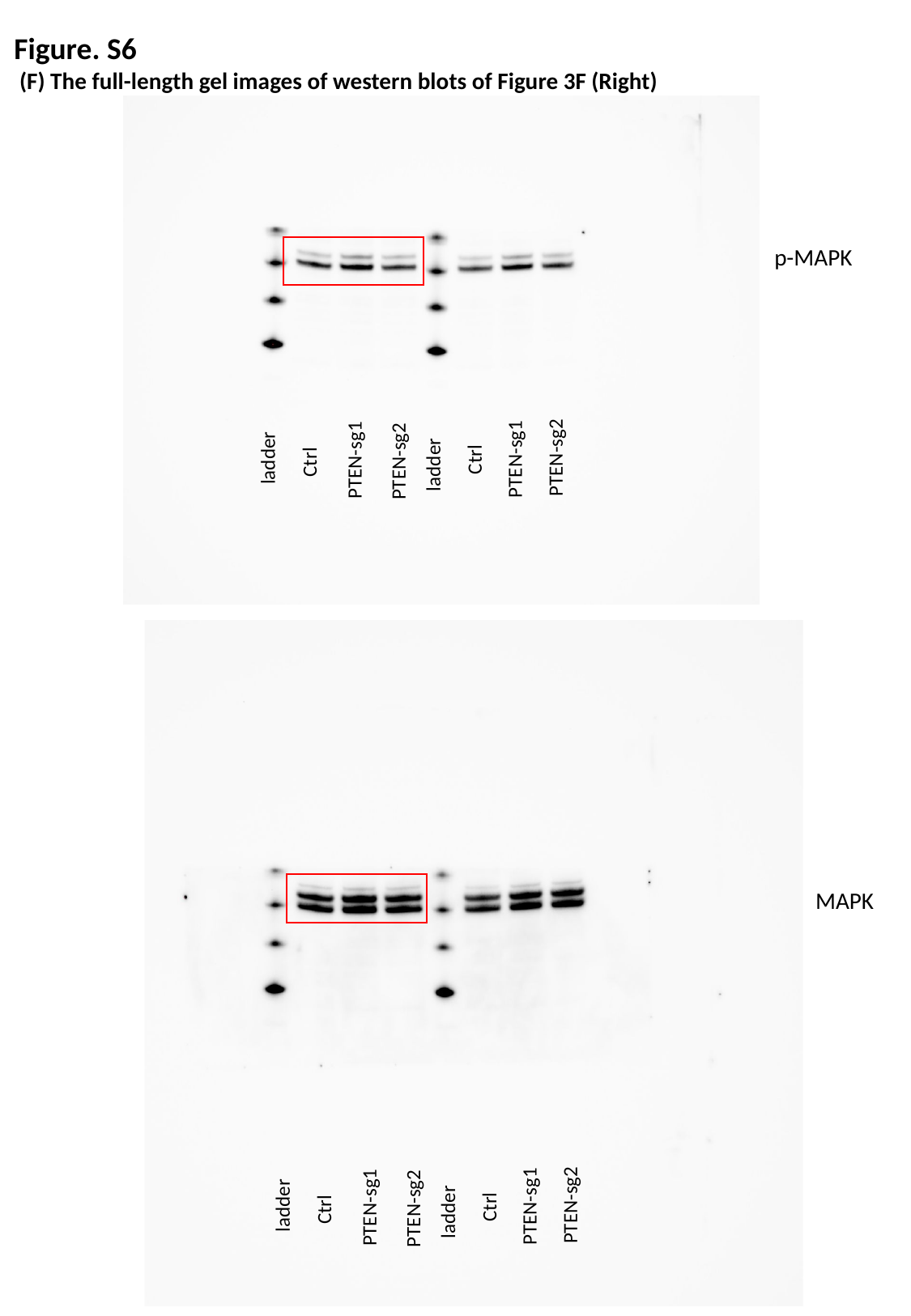

Figure. S6
(F) The full-length gel images of western blots of Figure 3F (Right)
p-MAPK
PTEN-sg2
ladder
PTEN-sg1
Ctrl
PTEN-sg1
PTEN-sg2
Ctrl
ladder
MAPK
PTEN-sg2
ladder
PTEN-sg1
Ctrl
PTEN-sg1
PTEN-sg2
Ctrl
ladder

## Slide 22
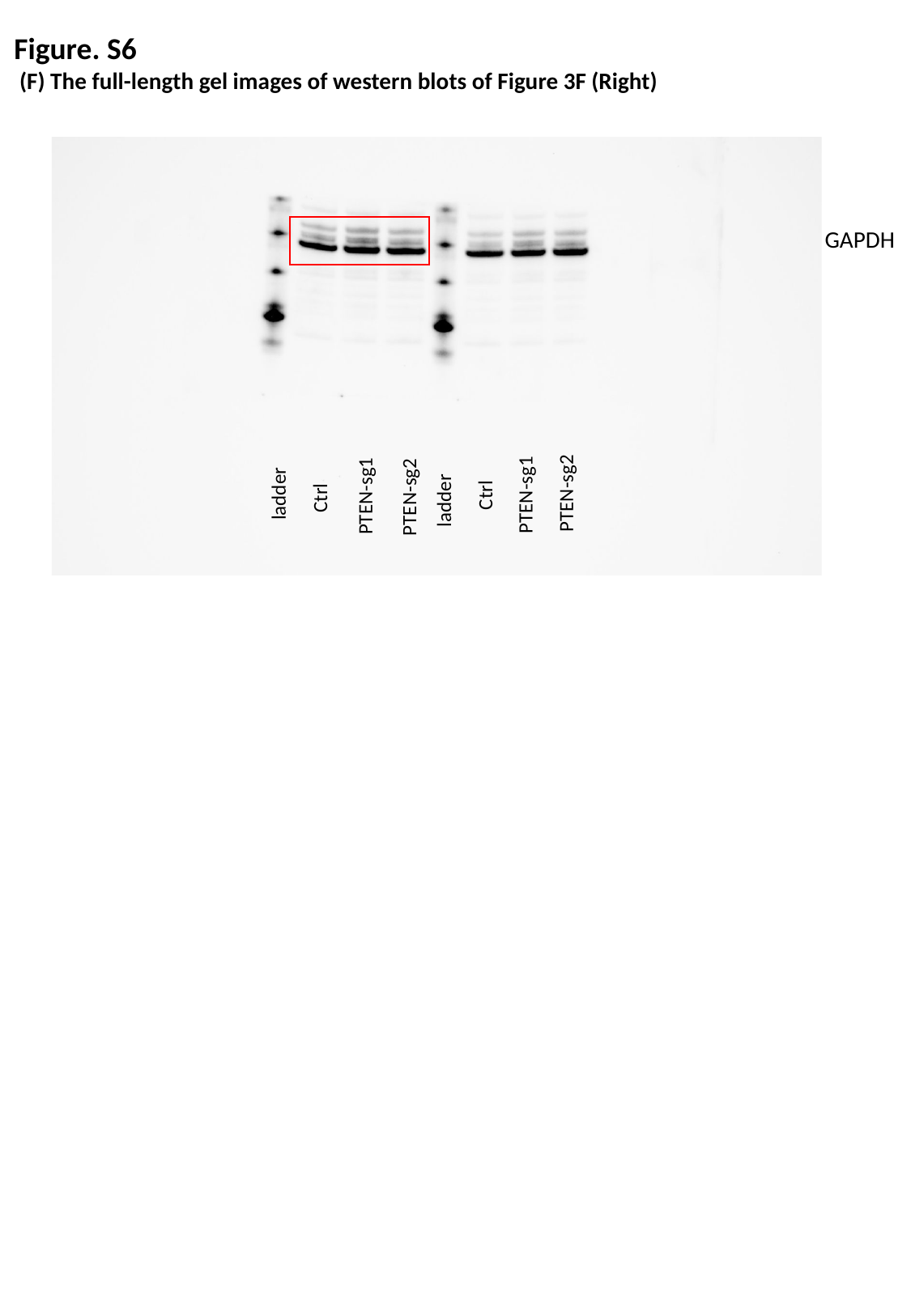

Figure. S6
(F) The full-length gel images of western blots of Figure 3F (Right)
GAPDH
PTEN-sg2
ladder
PTEN-sg1
Ctrl
PTEN-sg1
PTEN-sg2
Ctrl
ladder

## Slide 23
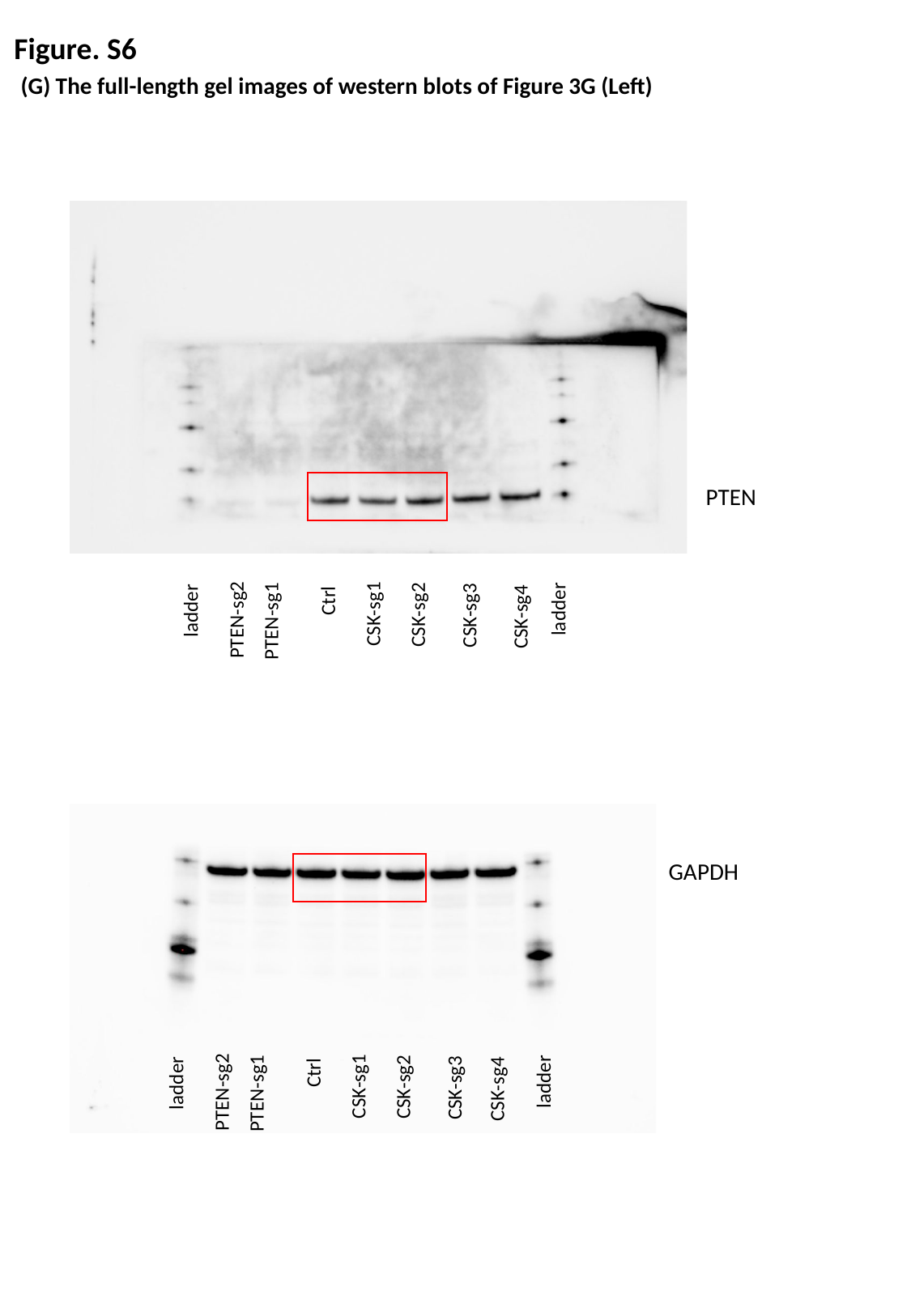

Figure. S6
(G) The full-length gel images of western blots of Figure 3G (Left)
PTEN
Ctrl
ladder
ladder
CSK-sg1
CSK-sg2
CSK-sg3
CSK-sg4
PTEN-sg2
PTEN-sg1
GAPDH
Ctrl
ladder
ladder
CSK-sg1
CSK-sg2
CSK-sg3
CSK-sg4
PTEN-sg2
PTEN-sg1

## Slide 24
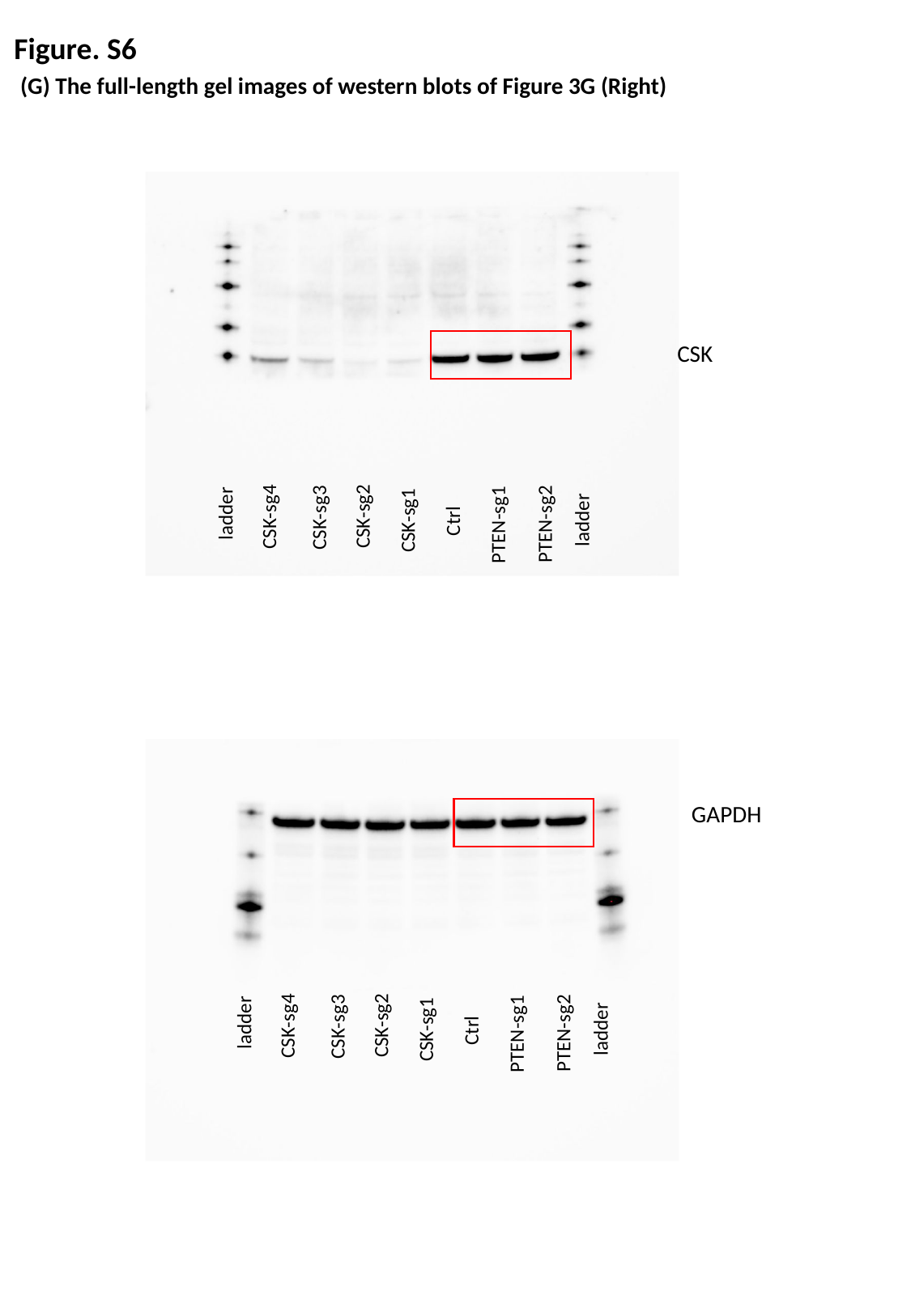

Figure. S6
(G) The full-length gel images of western blots of Figure 3G (Right)
CSK
CSK-sg3
ladder
CSK-sg2
CSK-sg4
CSK-sg1
ladder
Ctrl
PTEN-sg2
PTEN-sg1
GAPDH
CSK-sg3
ladder
CSK-sg2
CSK-sg4
CSK-sg1
ladder
Ctrl
PTEN-sg2
PTEN-sg1

## Slide 25
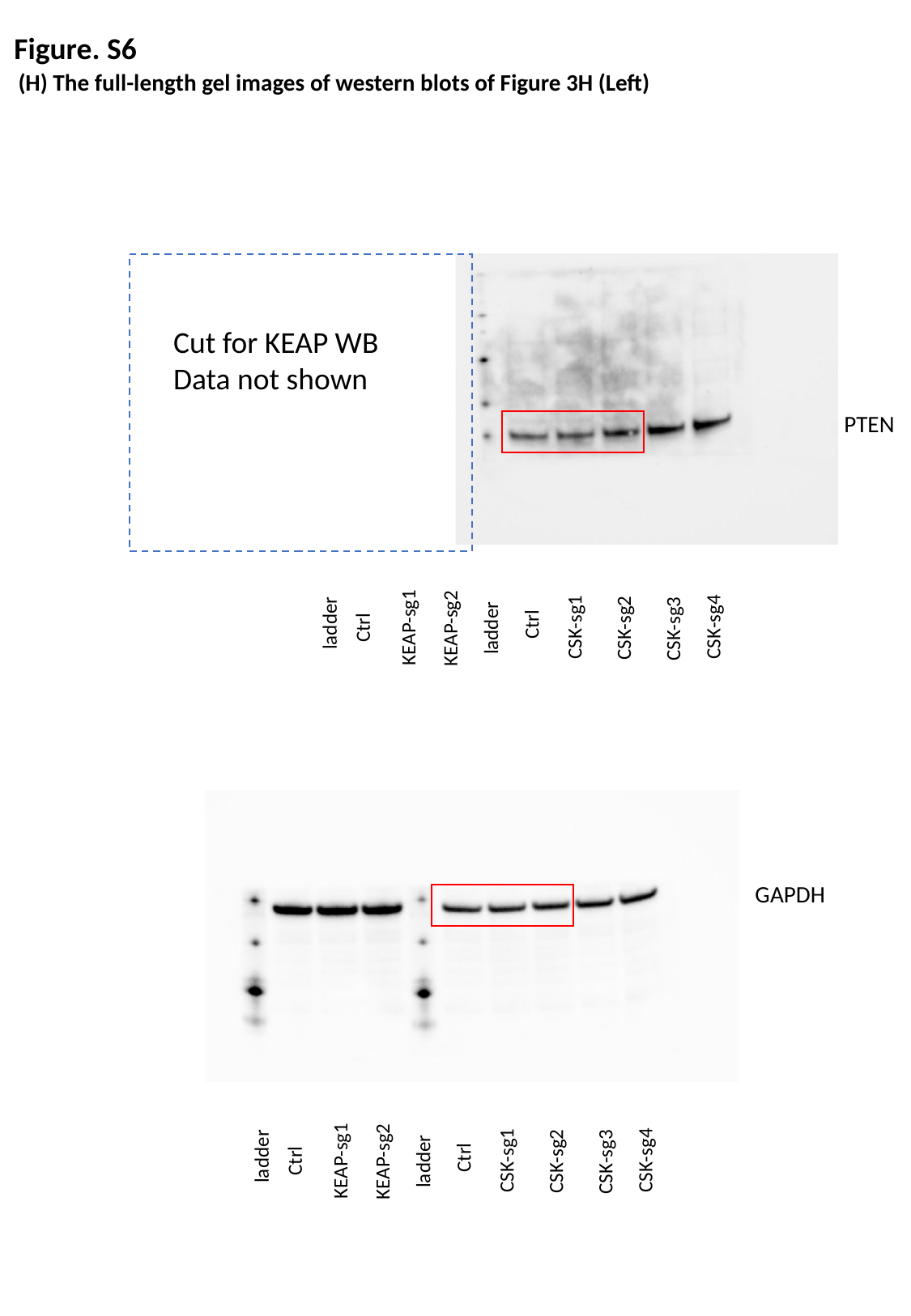

Figure. S6
(H) The full-length gel images of western blots of Figure 3H (Left)
Cut for KEAP WB
Data not shown
PTEN
ladder
Ctrl
CSK-sg4
CSK-sg1
KEAP-sg1
Ctrl
ladder
CSK-sg2
KEAP-sg2
CSK-sg3
GAPDH
ladder
Ctrl
CSK-sg4
CSK-sg1
KEAP-sg1
Ctrl
ladder
CSK-sg2
KEAP-sg2
CSK-sg3

## Slide 26
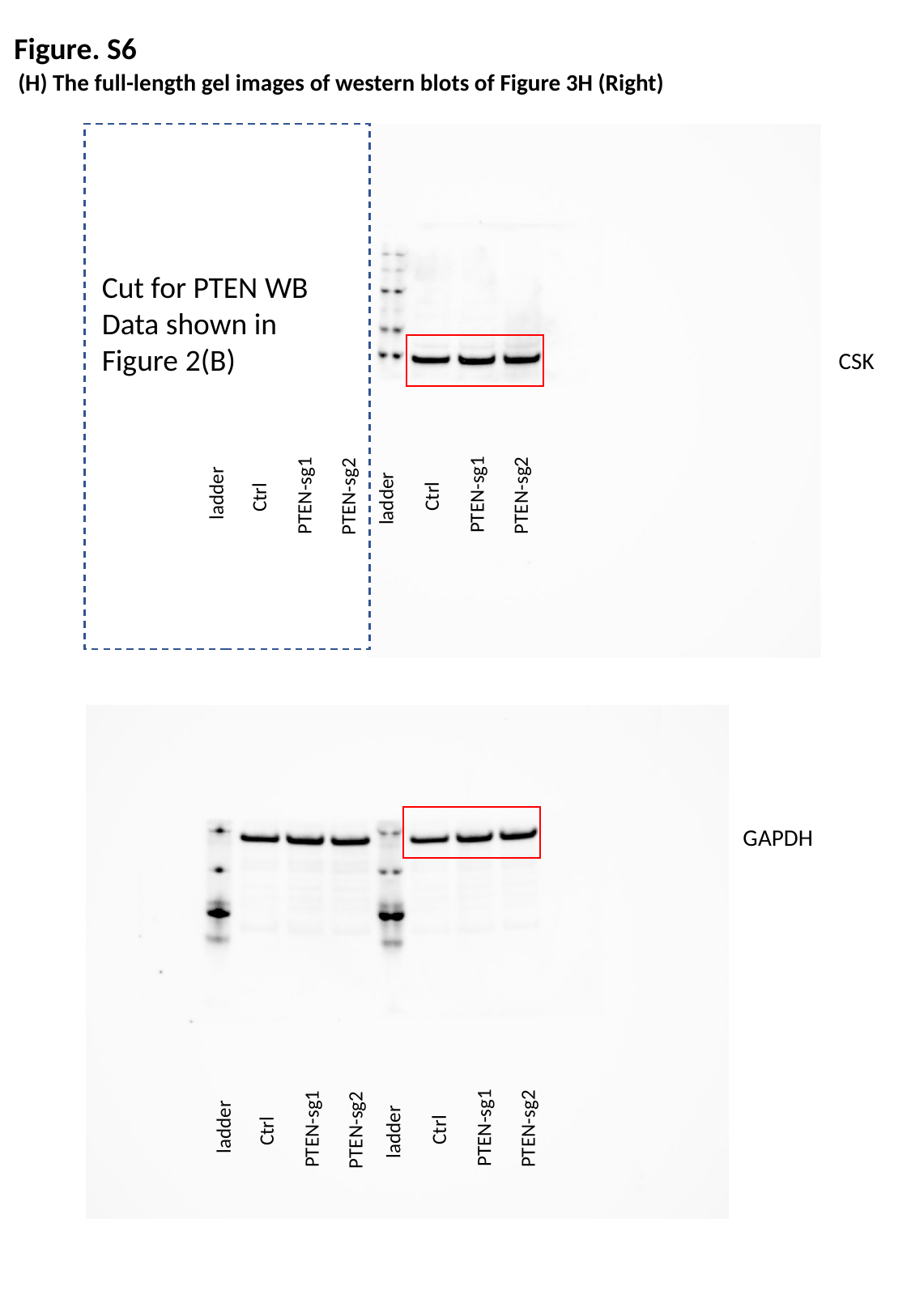

Figure. S6
(H) The full-length gel images of western blots of Figure 3H (Right)
Cut for PTEN WB
Data shown in Figure 2(B)
CSK
ladder
PTEN-sg1
PTEN-sg2
PTEN-sg1
Ctrl
PTEN-sg2
Ctrl
ladder
GAPDH
ladder
PTEN-sg1
PTEN-sg2
PTEN-sg1
Ctrl
PTEN-sg2
Ctrl
ladder
